# Neurons and astrocytes have distinct organelle signatures and responses to stress

**DOI:** 10.1016/j.celrep.2025.116280

**Published:** 2025-09-15

**Authors:** Shannon N. Rhoads, Weizhen Dong, Chih-Hsuan Hsu, Ngudiankama R. Mfulama, Ridhi Yarlagadda, Joey V. Ragusa, Michael Ye, Andy Henrie, Maria Clara Zanellati, Graham H. Diering, Todd J. Cohen, Sarah Cohen

**Affiliations:** 1 Neuroscience Center, University of North Carolina, Chapel Hill, NC, USA; 2 Cell Biology and Physiology, University of North Carolina, Chapel Hill, NC, USA; 3 Neurology, University of North Carolina, Chapel Hill, NC, USA; 4 DataTecnica LLC, Washington, DC, USA; 5 Lead contact

## Abstract

Neurons and astrocytes play critical yet divergent roles in brain physiology and neurological conditions. Intracellular organelles are integral to cellular function. However, an in-depth characterization of organelles in live neural cells has not been performed. Here, we use multispectral imaging to simultaneously visualize six organelles—endoplasmic reticulum (ER), lysosomes, mitochondria, peroxisomes, Golgi, and lipid droplets— in live primary rodent neurons and astrocytes. We generate a dataset of 173 *z* stack and 98 time-lapse images, accompanied by quantitative ‘‘organelle signature’’ analysis. Comparative analysis reveals a clear cell-type specificity in organelle morphology and interactions. Neurons are characterized by prominent mitochondrial composition and interactions, while astrocytes contain more lysosomes and lipid droplet interactions. Additionally, neurons display a more robust organelle response than astrocytes to acute oxidative or ER stress. Our data provide a systems-level characterization of neuron and astrocyte organelles that can be a reference for understanding cell-type-specific physiology and disease.

## In brief

Rhoads et al. demonstrate that neurons and astrocytes have distinct organelle compositions, morphologies, interactions, distributions, and dynamics. Through direct comparison between cell types, they find that these “organelle signatures” reflect known physiological functions and are remodeled following acute stress in a cell-type- and stress-specific manner.

## Graphical Abstract

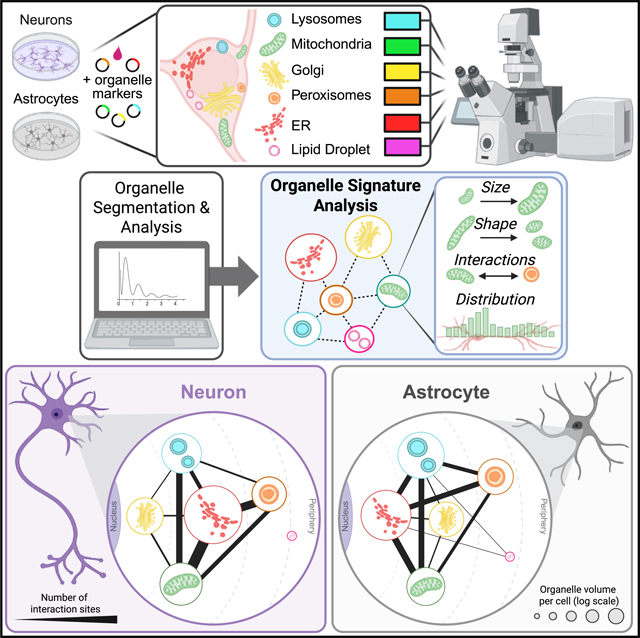

## INTRODUCTION

Multicellular organisms have gained an evolutionary advantage through differentiation of specialized cell types. Cells are further compartmentalized into organelles that carry out separate yet coordinated biochemical reactions. Organelles can provide insight into cellular physiology and function. This idea is exemplified in specialized cell types such as adipocytes and muscle cells, which contain unique organelle landscapes to sustain their dedicated functions. Adipocytes are composed of a central large lipid droplet (LD) for lipid storage and a comparatively smaller cytoplasmic region for other organelles ^1^ ; muscle cells contain an intricate mitochondrial reticulum for efficient metabolite diffusion during muscle contraction. ^2,3^ These examples illustrate the importance of a single organelle type to cell function. However, organelles do not operate independently. Organelle interactions at membrane contact sites and areas of close proximity occur to coordinate the activities of individual organelles. The contact sites are involved in signaling pathways and the transfer of ions, lipids, metabolites, and proteins. ^4^ Moreover, alterations in organelle morphology and interactions are integral to cell functionality. ^5–11^

Approaches to visualize multivariate phenotypes, such as simultaneous changes in organelle morphology and interactions, can be helpful in inferring organelle and cell state and function. However, techniques to study more than three to four organelles at a time are limited. Electron microscopy (EM) is the most common technique, but it requires harsh fixation protocols. Additionally, transmission EM has historically been limited to single thin slices. With the advent of focused ion beam-scanning EM, the field can now study volumetric samples, making the analysis of complex cell types, such as neurons, possible. ^12^ However, the imaging and analysis procedures are time-consuming and low-throughput. Recently, our group and others have utilized multispectral confocal microscopy for high-throughput, multi-organelle analysis. ^13–17^ This approach enables live-cell imaging of up to eight fluorescently labeled structures simultaneously with the speed and resolution of traditional confocal microscopy. Multispectral microscopy provides more accurate population estimations that take cell-to-cell variability into account and is compatible with robust, high-throughput analyses.

Spectral imaging can provide insight into organelle composition and inter-organelle communication networks. However, a global description of organelles in live primary brain cells, including neurons and astrocytes, has yet to be achieved. Neurons and astrocytes are morphologically and functionally distinct brain cells. Neurons are specialized to facilitate long-range electrochemical communication and endure over an organism’s lifetime. Astrocytes are a heterogeneous class of neuroglia with many known functions; they regulate synapses, maintain the blood-brain barrier, guide neuronal migration, and regulate immune responses to disease and injury.^18 ,19^ Proteomic characterizations of neurons and astrocytes reflect their functional specificity, revealing prominent ion-binding and synaptic signaling pathways in neurons and lipid metabolism and cell-cell interactions in astrocytes. ^20^ Moreover, in neurodegenerative diseases, including amyotrophic lateral sclerosis, Alzheimer’s disease, and Parkinson’s disease, pathogenesis is driven by unique functional deficits across multiple cell types, including neurons and astrocytes. These deficits include the dysregulation of diverse cellular processes, from protein quality control systems to mitochondrial dysfunction. ^21–23^ Characterizing the landscape of the major membrane-bound organelles involved in these processes holds promise in furthering our understanding of cell-type-specific functions in health and disease.

Examination of the endoplasmic reticulum (ER), Golgi (GL), and lysosomes (LS) can provide insight into the secretory pathway. ^24–27^ The ER is a primary site of lipid synthesis and protein folding; proteins are then sorted at the GL and degraded in LS. Mitochondria (MT), peroxisomes (PO), and LDs are metabolic organelles that play roles in lipid metabolism. ^28–30^ Additionally, MT generate ATP through oxidative phosphorylation, ^31^ while PO both generate and serve as a sink for reactive oxygen species (ROS).^32^ Multiple organelles, including ER, MT, and LS, also play important roles in calcium signaling. ^33^ Studying these organelles individually and in combination can paint a holistic picture of a cell’s inner workings.

Here, we present a dataset of three-dimensional (3D) single time point and two-dimensional (2D) time-lapse confocal microscopy images containing six simultaneously labeled organelles—ER, LS, MT, PO, GL, and LD—in commonly used primary rodent neuron and astrocyte culture models. The 3D images allow quantitative, single-cell analyses of organelle morphology, inter-organellar interactions, subcellular distribution, and cell morphometrics. Our analysis approach yielded 1,418 metrics per cell. Time-lapse images provided further insight into organelle dynamics. Here, we showcase a curated set of metrics to summarize the organelle landscapes of neurons and astrocytes and their responses to acute stress. We highlight key distinctions between cell types and conditions.

## RESULTS

### Organelle signatures describe the morphology, interactions, and distribution of organelles at the single-cell level

**Figure 1.**
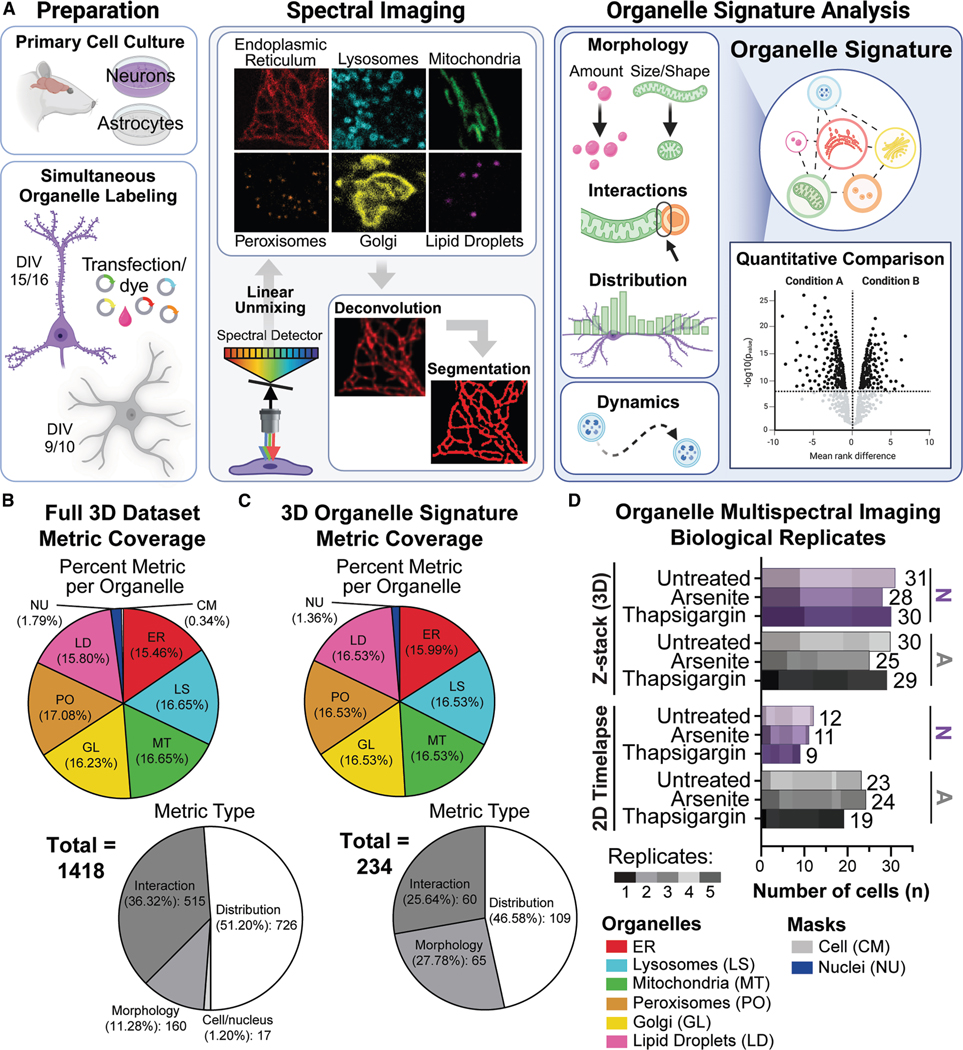
Comparative analysis of neuron and astrocyte organelle signatures (A) Experimental design: in independent cultures of neurons (purple) and astrocytes (gray), six organelles were simultaneously fluorescently labeled (left) and imaged by multispectral microscopy (center). Organelle objects were segmented from 3D images (center) and quantified to produce the organelle signature (right). Parallel 2D time-lapse microscopy images were quantified to reveal organelle dynamics (right). (B and C) Organelle coverage and measurement types for the full 3D dataset (B) and the curated “organelle signature” dataset used in initial phenotypic screens (C). (D) Biological replicates of the neuron (“N”) and astrocyte (“A”) multispectral imaging datasets used throughout Figures 2, 3, 4, 5, 6, and 7. Also, see Figure S1.

**Figure 2.**
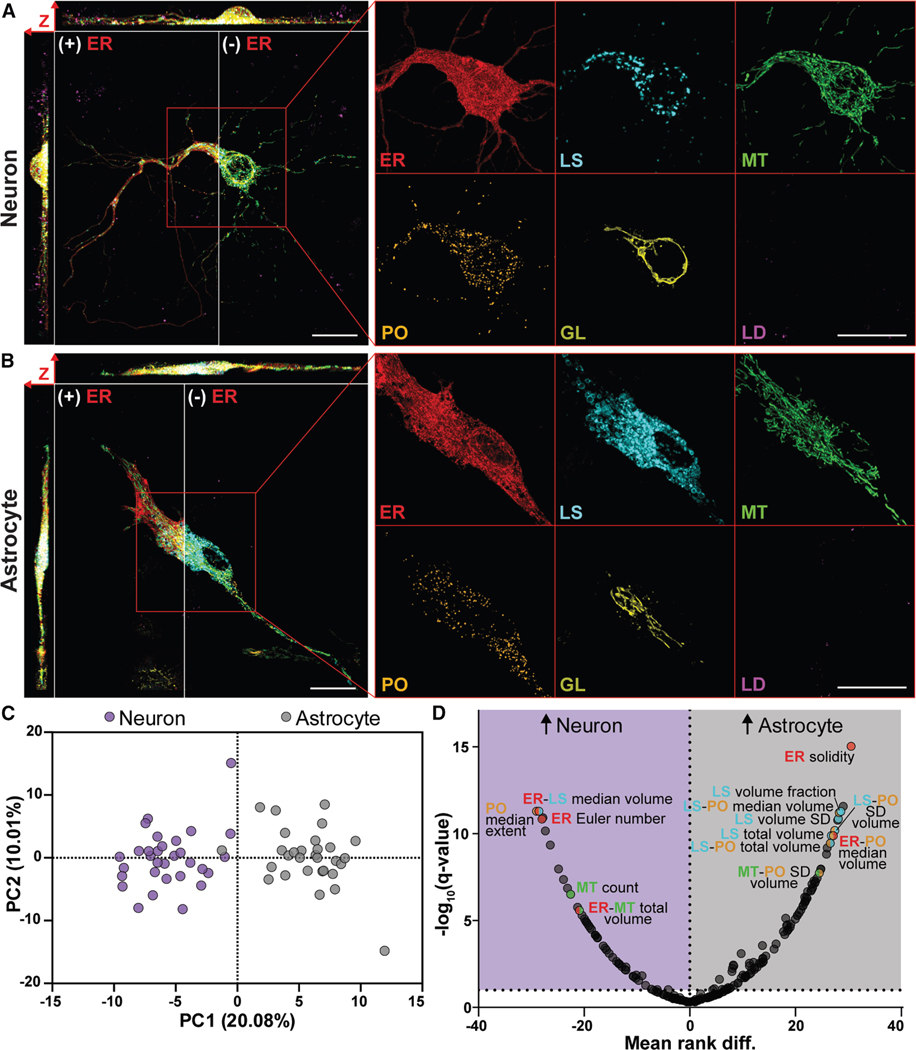
Neurons and astrocytes display cell-type-specific organelle signatures (A and B) Maximum intensity projections of multispectral image *z*-stacks of a neuron (A) and astrocyte (B) labeled for the endoplasmic reticulum (ER), lysosomes (LS), mitochondria (MT), peroxisomes (PO), Golgi (GL), and lipid droplets (LD). Scale bars, 20 μm. (C) Principal component (PC) scores of neurons and astrocytes based on PC analysis of 3D organelle signatures; percentages represent variance explained by the PC. (D) Volcano plot of 3D organelle signature metrics (234 metrics) between neurons and astrocytes; statistical significance was determined by a 10% false discovery rate. Statistical outcomes are explored in more detail in Figures 3, 4, and 5. SD, standard deviation. (C) and (D) Sample size and replicate information are summarized in Figure 1D. Also, see Figure S2 and Table S1.

We set out to characterize the variation and responses of organelles that underlie cell-type differences in neurons and astrocytes. We utilized quantitative multispectral microscopy of organelles in independent cell culture models (Figures 1 and S1). ^17,34^ Both cell types were obtained from Sprague-Dawley rats, cultures were purified to produce monocultures, and neurons were maintained through synaptogenesis (day *in vitro*; DIV15–16; Figure 1, left). ^35^ Neuron cultures contained a majority (91.49%) of NeuN-positive cells (Figure S1A); 84.31% were excitatory (TBR1+) and 15.69% were inhibitory (GAD1+; Figure S1B). Neurons or astrocytes were simultaneously labeled with fluorescent markers specific to the ER, LS, MT, PO, GL, and LD one day prior to imaging (Figure 1A, left; Figure S1C). We utilized a confocal microscope adapted with a spectral detector to visualize the six organelles simultaneously via multispectral microscopy (Figure 1A, center). At the time of imaging, the organelle labeling process had a minor impact on cell viability and no detectable activation of ER stress (Figures S1D and S1E). Upon visual inspection, neurons displayed canonical pyramidal shapes without neuritic blebbing or beading, ^36,37^ astrocytes were elongated with several fine processes, ^38^ and nuclei displayed normal morphologies (Figures 2A and 2B; Video S1). ^39^ Both single-timepoint *Z*-stacks (3D) and single-Z-plane (2D) time-lapse images were acquired to map the 3D structure of organelles and their dynamics, respectively. All spectral images were processed by linear unmixing, as previously reported, ^17,34^ to produce six-channel images (Figure 1A, center). The images were then deconvolved (Figures S2A and S2B). For 3D images, instance segmentation of organelles was performed on each intensity channel separately (Figures S2A and S2B). Cell and nucleus masks were also inferred from a combination of intensity channels. The 2D time-lapse images were not segmented prior to analysis.

To quantitatively compare cell types and cellular conditions (Figure 1A, right), segmented organelle objects from 3D images were assessed independently for aspects of organelle morphology and subcellular distribution. Then, they were considered in pairwise combinations to quantify organelle interactions. The cell and nuclei masks were also measured for aspects of cellular morphology. Together, these analyses resulted in 1,418 metrics per cell (Figure 1B). We utilized a curated subset of 234 metrics, herein referred to as the “organelle signature,” to screen for differences between experimental conditions using statistical analysis (Figure 1C). The curation process minimized metric redundancy and ensured equal coverage across each measurement type and organelle. The differences between organelle signatures were further explored using additional metrics from the full 3D dataset and independent time-lapse analyses.

The quantitative multispectral microscopy datasets described herein include two cell types—neurons and astrocytes—and three cellular conditions each: vehicle-treated controls (“controls”) and two acute drug exposures to induce oxidative or ER stress, conditions which perturb the system in a manner associated with neurological disorders where neuron and astrocyte dysfunction are observed. ^22^ The sample sizes and biological replicate information for multispectral images and analyses are included in Figure 1D.

### Neurons and astrocytes display cell-type-specific organelle signatures

We first compared the control neurons and astrocytes to establish differences in their baseline organelle signatures. Representative images of control cells and example segmentations are included in Figures 2A, 2B, S2A, and S2B. We gained a system-wide perspective on organelle signature differences using principal component analysis (PCA). Principal component (PC) 1 scores clearly distinguished between the two cell types (Figure 2C). Independent PCA of the neuron and astrocyte organelle signatures separately also detected possible subpopulations in both cultures (Figures S2C and S2D). Two cells from the neuron cultures had a higher PC1 or a lower PC2 value, suggesting the possibility of two distinct neuron subpopulations that could align with the ∼15% inhibitory and/or ∼9% NeuN-negative cells observed in our cultures (Figures S1A and S1B). The astrocytes exhibited a slightly bimodal separation along PC2, which could reflect functional or metabolic heterogeneity in the astrocyte cultures. However, the magnitude of separation between cells of the same type was far smaller than the separation between neurons and astrocytes.

To elucidate which metrics were significantly different between cell types, we conducted a Mann-Whitney U test with multiple comparisons (Figure 2D; Table S1). A false discovery rate of 10% resulted in 144 metrics that were significantly different between cell types, while 90 metrics showed no significant difference. We also compared the organelle signatures of control neurons cultured in astrocyte-conditioned media with those of astrocyte-naïve neurons. The astrocyte-conditioned neurons showed evidence of astrocyte trophic support,^40–42^ including differences in cell and nucleus shape metrics from the full 3D dataset (Figure S1F) and an increase in branching within the proximal dendrites (Figures S1G and S1H). ^40–42^ PCA of the curated organelle signature metrics revealed no obvious separation between the two neuron conditions (Figure S2E), and the Mann-Whitney U test found only seven significantly different metrics (Table S1). This suggests that the indirect transfer of extracellular materials from astrocytes to neurons can impact neuron organelle signatures, but only minimally compared to cell type differences.

These findings support the conclusion that organelle signatures can be a useful tool for understanding differences in cellular physiology across cell types and environmental conditions.

### Organelle abundance relates to neuron and astrocyte functions

**Figure 3.**
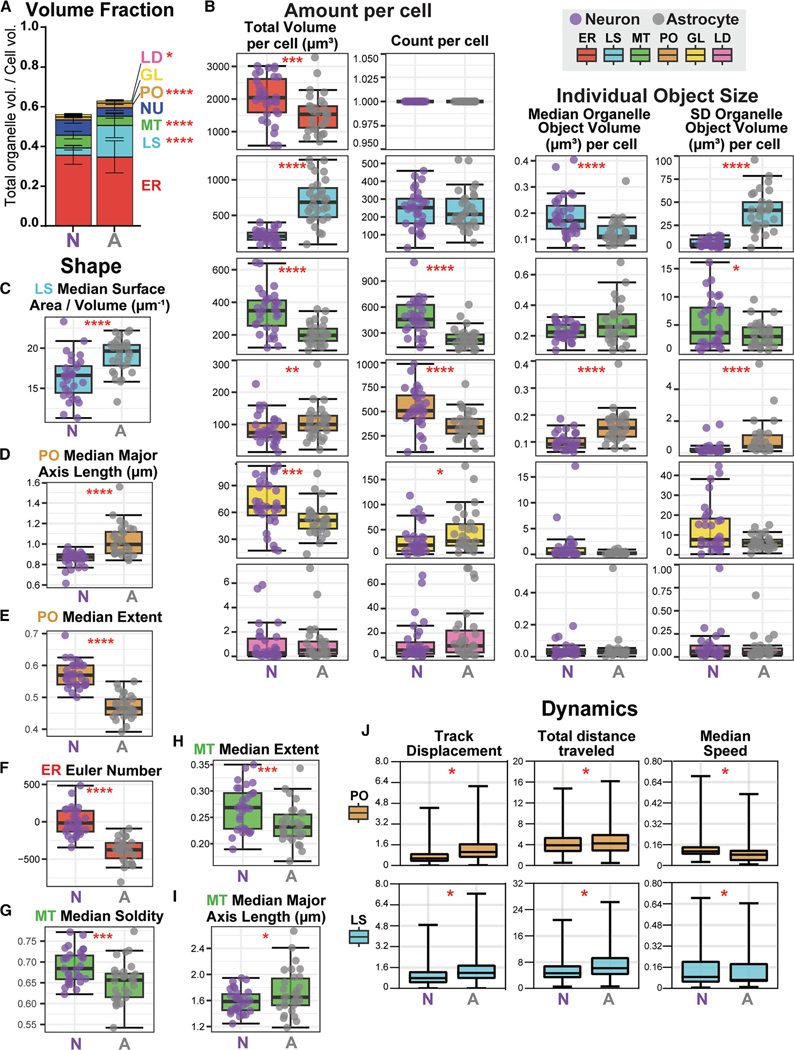
Organelle morphology and dynamics are unique between cell types (A–I) 3D organelle signature metrics describing the salient organelle morphology—amount, size, and shape—differences between neurons (“N”) and astrocytes (“A”). Volume (vol.) fraction (A), organelle count (B), size (B), and shape (C–I) metrics for the endoplasmic reticulum (ER), lysosomes (LS), mitochondria (MT), nucleus (NU), peroxisomes (PO), Golgi (GL), and lipid droplets (LD) per cell. The ER is constrained to a single object during segmentation, resulting in a uniform ER count per cell. Salient shape metrics include median surface area-to-volume ratio (C), median major axis length (D and I), median extent (E), Euler number (F), and median solidity (G). Bar charts depict mean ± standard deviation (SD), and boxplots depict median, interquartile range, and range. Asterisks denote *q* values from Figure 2D and Table S1; sample size and replicate information are summarized in Figure 1D. Data points represent single cells. (J) Boxplots of organelle dynamics. Values reflect population summaries across multiple cells; boxplots depict median, interquartile range, and range; asterisks represent *p* values from independent analyses for each organelle separately. Also, see Figure S3 and Table S1.

We included 65 organelle morphology metrics (amount, size, and shape) in our curated 3D organelle signature analysis (Figure 1C); 34 differed significantly between neurons and astrocytes (Table S1). We chose organelle volume fraction, total volume, and count (Figures 3A and 3B) to assess the amount of each organelle per cell. The volume fraction is the fraction of the cell volume occupied by each organelle type. The proportion of cell volume allotted to each organelle could shed light on the relative importance of that organelle’s function to cellular homeostasis. In contrast, the total volume and count represent the absolute amount of each organelle without consideration of cell size; they could indicate the total capacity of that cell to carry out organelle-specific functions. All six organelles differed in one or more of these amount metrics between neurons and astrocytes (Figures 3A and 3B). Generally, neurons contained a higher total volume of ER, MT, and GL, while astrocytes were higher in LS and PO (Figure 3B). The total volume of LD did not differ between cell types, but the volume fraction was higher in astrocytes (Figures 3A and 3B). Western blotting and previously published RNA sequencing data of canonical organelle proteins corroborated these trends (Figures S3A and S3B).^43 ,44^

The most striking difference in organelle amount was a higher LS volume fraction and total volume in astrocytes than in neurons (Figures 3A and 3B). Supporting our findings, the canonical LS proteins LAMP1 and Rab9 were reported to have higher transcript levels in astrocytes (Figure S2A). ^43,44^ These data imply that astrocytes have a higher lysosomal capacity and require more lysosomal activity for homeostasis than neurons. Conversely, higher MT volume fraction, total volume, and count in neurons indicate that mitochondrial functions, such as ATP production through oxidative phosphorylation (OXPHOS), may be needed at a higher capacity to achieve proper neuronal homeostasis. Consistent with this observation, transcripts for the inner mitochondrial membrane protein and respiratory chain component COX5B and the outer mitochondrial membrane protein TOM20 were reportedly higher in neurons (Figure S3A). ^43,44^ Furthermore, immunoblotting showed that TOM20 protein levels were significantly higher in neurons (Figure S3B).

### Organelle morphology and dynamics are unique between cell types

Our curated 3D organelle signature analysis also included measurements of organelle object size—individual object volume and standard deviation (SD) of object volumes per cell—and shape—surface area-to-volume ratio, equivalent diameter, extent, Euler number, solidity, and major axis length. The median value per cell for each metric, except the SD of volumes per cell, was included in the organelle signature analysis. We identified nuanced differences in the size and shape of each organelle between cell types except LD (Table S1; Figures 3C–3I), which were spherical and relatively low in abundance.

In astrocytes, the LS population had a lower median object volume but a higher SD of volumes per cell (Figure 3B). Because segmented organelle objects in our analysis can represent closely clustered groups of organelles as well as individual organelles, these metrics indicated that astrocytes either contained a wide range of relatively spherical LS objects or a variety of different-sized LS clusters. To better understand which phenotype was occurring, we examined differences in the LS object shape. Most notably, the median LS surface area-to-volume ratio was higher in astrocytes (Figure 3C). Since the surface area-to-volume ratio of a sphere reduces as volume increases, it is more likely that astrocytes contain clusters of LS. The example images confirmed these findings (Figures 2A and 2B). We also noted that individual LS in intensity images appeared larger in astrocytes than in neurons.

Distinct from LS, the PO population in neurons had a higher count and a lower median and SD of volumes per cell (Figure 3B). This represents a smaller but more numerous PO phenotype in neurons. We also observed that most of the PO shape measurements differed between cell types (Table S1). In particular, the PO major axis length was higher in astrocytes (Figures 3D and S3C), suggesting that astrocytes have longer PO objects than neurons. We then examined the PO extent to better understand if the objects were more spherical or elongated (Figure S3D). The median extent value was higher in neurons (Figure 3E), indicating that the objects were more spherical. Representative images in Figure 2 exemplify these phenotypes, showing that neurons contained more numerous but smaller PO that were not clustered into groups, while astrocytes had larger PO objects that would occasionally form small, elongated clusters (Figures 2A, 2B, S2A, and S2B). Interestingly, PEX11β, a peroxisomal protein with higher transcript levels in neurons (Figure S3A), ^43,44^ was reported to increase peroxisomal proliferation rates. ^45,46^ This could explain the presence of more numerous, smaller peroxisomes in neurons. A reduction of PEX11β has also been implicated in peroxisomal clustering. ^47^ Lower PEX11β transcript levels could indicate the role of this protein in astrocyte PO clustering.

In conjunction with 3D organelle signature analysis, we also compared the dynamics of round organelles from 2D time-lapse images. The LS and PO, organelles that traffic along microtubules using shared machinery, ^48,49^ showed differences in their dynamics between cell types (Figure 3J). We observed a higher track displacement and distance traveled and lower speed for both organelles in astrocytes, indicating that the organelles moved in a more directed but slower manner. The plots of the organelle tracks display these differences, showing that astrocyte organelles cover larger net distances from the origin (track start; Figures S3G and S3H). ^48,49^ These findings suggest that neurons and astrocytes differentially regulate microtubule-based trafficking of organelles.

### Some organelle morphology phenotypes correlate with cell morphology

Size differences were also evident in more morphologically complex organelles such as the ER and MT (Table S1). The Euler number, a measure of object topology that considers the number of holes within and tunnels through an object (Figure S3E), is higher in neurons (Figure 3F). This finding indicates that the ER in neurons contains fewer tunnels than in astrocytes, likely reflecting crowding in the relatively small soma and neurites of neurons. Moreover, neuronal MT had a higher median solidity and extent and lower major axis length than astrocytic MT (Figures 3G–3I and S3F). Together with the higher SD of MT volumes in neurons (Figure 3B), these metrics indicate a wider range of MT sizes in neurons, possibly including more fragmented MT.

Because these objects, especially the ER, can create a connected network throughout the cell, we assessed the correlation between cell and organelle morphology in both cell types (Figure S3I; Table S2). Some organelle metrics, including the count and total volume of LS, MT, PO, and ER, scaled with cell size (e.g., positive correlation with volume, equivalent diameter, etc.) in neurons and astrocytes. However, most organelle shape metrics had much weaker correlations with cell size or shape, and some even had negative correlations. Notably, the ER equivalent diameter and extent correlated with several cell metrics, including cell volume, equivalent diameter, and extent. This finding was not surprising since the ER network traverses most of the cell volume and indicates that ER equivalent diameter and extent differences between cell types are likely due to differences in the neuron and astrocyte cell shape, not organelle shape.

### Distinct organelle interactomes and dynamics reflect functional and metabolic differences in neurons and astrocytes

Next, we examined organelle interactions in neurons and astrocytes by quantifying the amount and size of regions of overlap between pairwise combinations of organelles, herein known as organelle interaction sites (Figure S4A, see STAR Methods). These sites included membrane contact sites (10–80 nm) and areas of close proximity (<410 nm). Although this method of detection cannot determine whether an interaction site is due to membrane contact sites or other types of physiological interactions, such as co-trafficking, our group has previously shown that interaction site measurements correlate with the amount of *bona fide* organelle contacts detected using dimerization-dependent membrane contact biosensors.^50^ The total volume, count, median object volume, and SD of volumes per cell for all 15 pairwise interaction sites were included in our curated analysis (Table S1), resulting in 60 interaction metrics (Figure 1C). Together, these metrics define the organelle interactome.

**Figure 4.**
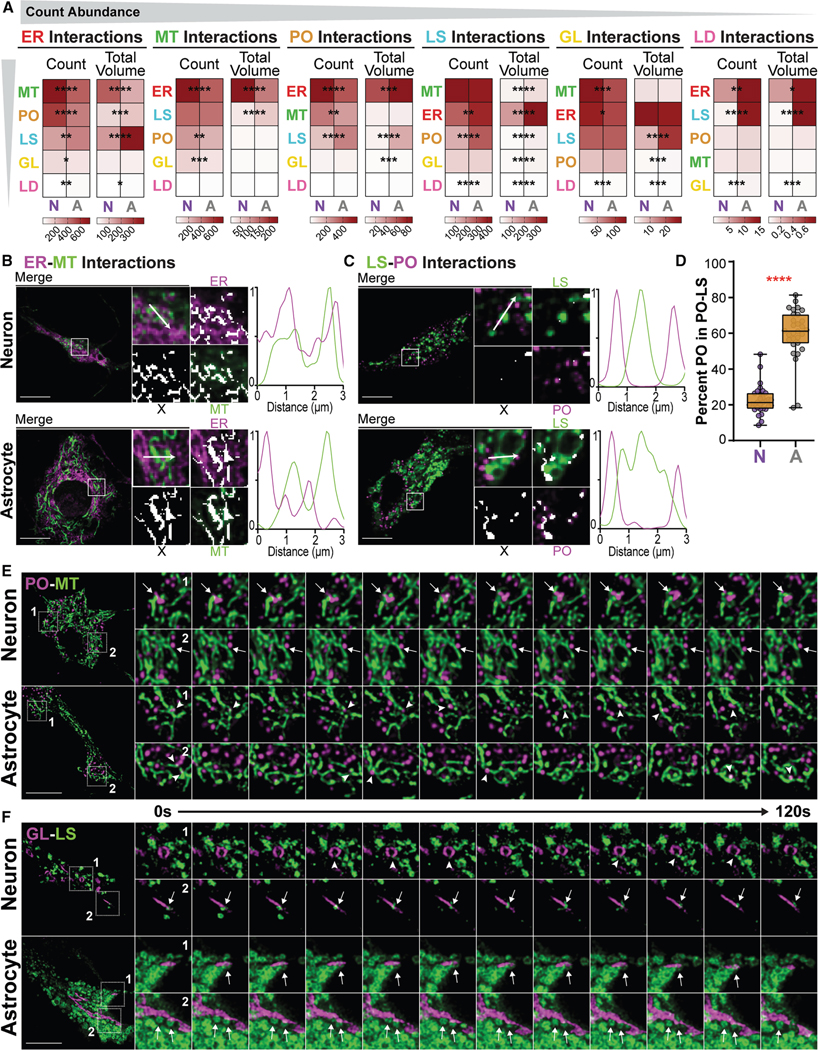
Distinct organelle interactomes and interaction dynamics reflect functional and metabolic differences in neurons and astrocytes (A) Heatmaps displaying the average count and volume (μm^3^) of pairwise organelle interaction sites per cell listed in order of count abundance; asterisks denote *q* values from Figure 2D; sample size and replicate information are summarized in Figure 1D. (B and C) Representative intensity images and segmented interaction sites (X, white) between ER-mitochondria (ER-MT) (B) and lysosome-peroxisome (LS-PO) (C) in neurons (top) and astrocytes (bottom). Scale bars, 10 μm. Line scan graphs display the normalized intensity measured across the white arrows. (D) Boxplot of the percentage of peroxisome (PO) interacting with lysosomes (LS), a metric included in the full 3D dataset. Data points represent single cells, and asterisks denote *p* value determined by independent t test; boxplots depict median, interquartile range, and range; sample size and replicate information are summarized in Figure 1D. (E and F) Representative frames from 2D time-lapse images showing peroxisome-mitochondria (PO-MT) (E) and Golgi-lysosome (GL-LS) (F) interactions every 10 s. Scale bars, 10 μm. Arrows indicate constant interactions over the 5-min imaging duration; arrowheads indicate transient interactions. LD, lipid droplet. Also, see Figure S4 and Table S1.

Based on interaction site counts, the relative abundance of organelle interactions was similar between cell types: ER interactions were the most abundant, followed by MT, PO, LS, GL, then LD (Figure 4A). As expected, the highest-volume organelle, ER, and the lowest-volume organelles, GL and LD, had the highest and lowest total interaction volumes, respectively (Figure 4A). Of note, most but not all pairwise interaction site volumes correlated with their constituent organelle volumes (Figure S4B). Interactions involving GL and LD often did not correlate with the amount of organelle they were interacting with. For example, LS-LD interaction volumes did not correlate with LS volume but did correlate with LD volume. This could be because LD and GL have less available surface area to facilitate the interactions compared to all other organelles (Figure S4C). Nonetheless, we were able to identify 42 organelle interaction metrics that differed between neurons and astrocytes, likely reflecting their differing cellular functions and metabolism (Table S1).

Neurons contained more ER-MT interactions with significantly higher metrics of amount and size compared to astrocytes (Figure 4A; Table S1). Within the neuron ER-MT interaction sites, the organelles appeared more closely intertwined, indicated by the regions of overlap (white) in the merged intensity images or as shared area under the intensity curves in the example line scan (Figure 4B). Western blotting revealed that the ER-MT tether proteins PTPIP51 and VAPB were more highly expressed in neurons (Figure S4D). PO-MT, MT-GL, and ER-GL interactions were also more common in neurons, but differed in size (Figure 4A; Table S1). These differences could indicate nuances in organelle co-regulation. However, the implications of different organelle interaction site sizes and shapes are not yet well defined.

Astrocytes had more prominent lysosomal interactions. For example, LS-PO and LS-LD interactions were higher in all four measurements of interaction amount and size (Table S1). Within the astrocyte LS-PO interaction sites, PO appeared closer to LS, as exemplified by the distance between peaks in the line scan analysis (Figure 4C). This finding led us to examine the PO-LS more closely. In the larger dataset of 1,418 metrics, the percentage of PO objects involved in LS-PO interactions was significantly higher in astrocytes (Figure 4D). We also found higher levels of LS-LD interactions in astrocytes; however, in our conditions, neurons had an average LD count of 12.29, compared to astrocytes with 18.53 (Table S1), making diyfferences in LD interactions statistically more likely in astrocytes and, therefore, challenging to interpret.

Leveraging time-lapse images, we further explored two interaction types: PO-MT and LS-GL. PO in neurons regularly maintained interactions with MT over the duration of the imaging period (Figure 4E, arrows; Video S2), while PO only transiently interacted with MT in astrocytes (Figure 4E, arrowheads; Video S2). This suggests that neurons contain more numerous PO-MT interaction sites due to the higher retention rate of the interactions over time. LS-GL interactions, which were higher in total volume, median volume, and SD of volumes in astrocytes (Figure 4A; Table S1), were sustained throughout the imaging period in both neurons and astrocytes (Figure 4F, arrows; Video S3). However, in neurons, LS-GL interactions occurred less frequently and seemed to happen preferentially at specific points along the GL objects. Transcripts for the tether proteins Rab34 and FLCN, which engage to form LS-GL membrane contact sites through the effector protein RILP,^51^ were reportedly higher in astrocytes, corroborating our findings (Figure S4E).^43 ,44^ In astrocytes, we also qualitatively observed reduced LS motility in regions surrounding the GL where LS-GL interactions occurred regularly, as previously reported.^51,52^

### Neuron and astrocyte organelles have distinctive *xy* and *z* distributions

**Figure 5.**
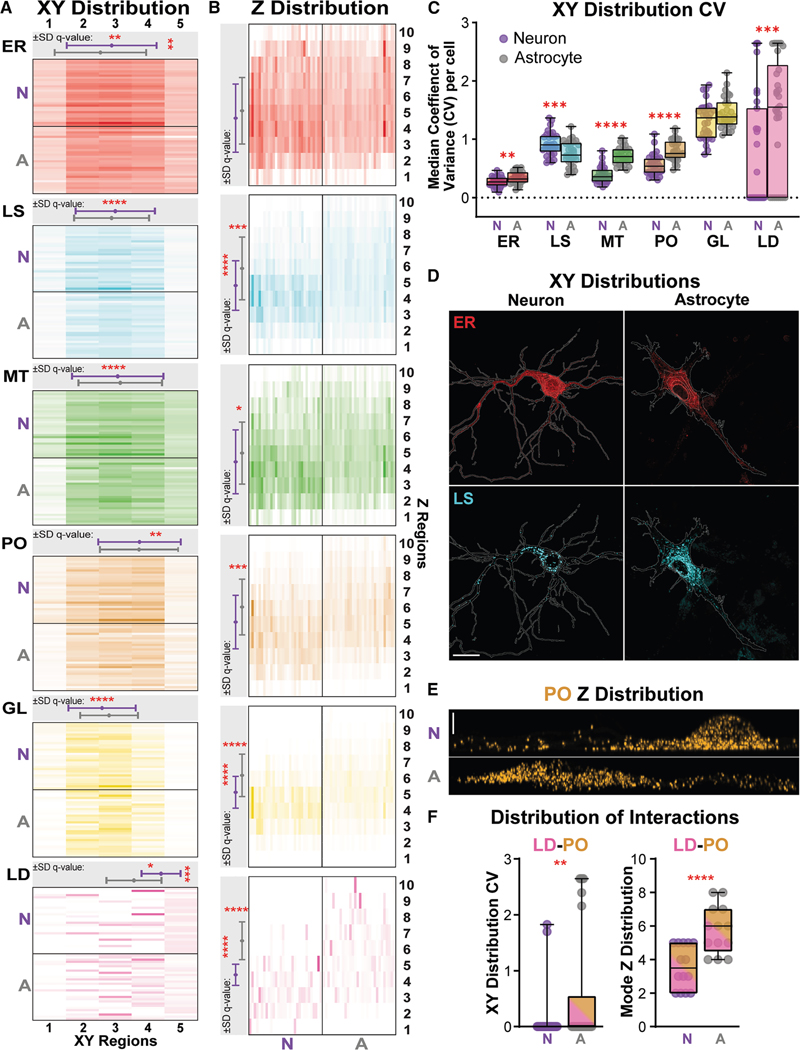
Neuron and astrocyte organelles and interaction sites have distinctive *xy* and *z* distributions (A–C and F) Organelle (A–C) and interaction site (F) distribution measurements per cell; asterisks denote *q* values from Figure 2D; sample size and replicate information are summarized in Figure 1D. A-B. Heatmaps display the normalized organelle volumes from the nucleus to the periphery (1–5; A) and from the bottom to the top (1–10; B) of the cell; each row (A) and column (B) represents the normalized volume values for individual cells. Dot plots above (A) and to the left (B) of the heatmaps summarize the mode (data point; asterisk perpendicular to the error bars) and SD (error bars; asterisk parallel to the error bars) distribution metrics from the 3D organelle signature analysis. Boxplots of the *xy* distribution median coefficient of variance (CV) (C) and distribution metrics for organelle interactions that were not correlated to their constituent organelles’ distributions (F); boxplots depict median, interquartile range, and range and data points represent single cells. (D) Maximum projections of endoplasmic reticulum (ER) and lysosome (LS) intensity images overlayed on the *xy* distribution regions (outlines). Scale bars, 20 μm. (E) Maximum projections of peroxisome (PO) intensity images; image orientation displays the *xz* axes; Scale bars, 5 μm. MT, mitochondria, GL, Golgi, and LD, lipid droplets. Also, see Figure S5 and Table S1.

The subcellular distribution of organelles has been shown to impact organelle function. ^53^ Here, we profiled and compared the subcellular distribution of organelles and organelle interactions in neurons and astrocytes (Figure 5). Our curated analysis included 109 metrics from subcellular distribution measurements in perinuclear to peripheral (*xy*) and *z*, independently, for all six organelles, 15 pairwise interaction sites, and the nucleus (Figure 1C). The *xy* distribution describes the spread of organelles from the nucleus to the cell membrane, while the *z* distribution quantifies the spread of organelles from the bottom to the top of the cell (Figure S5A; see STAR Methods).

The *xy* distribution of organelles was similar between neurons and astrocytes (Figure 5A). The *xy* region with the highest normalized organelle volume (mode) and the spread of the organelles across the five *xy* regions (SD) were stereotyped per organelle (Figure 5A, gray box): GL was perinuclear; ER, MT, and LS were centrally localized between the nucleus and plasma membrane; and PO and LD were more peripheral. Notably, subtle yet significant differences in ER and LD distributions were seen between cell types. ER and LD were more peripherally localized, with a lesser SD in neurons. Additionally, the ER and LD coefficient of variance (CV) values were lower in neurons (Figure 5C), indicating more consistency in volumes across each of the five concentric *xy* regions. Though less exaggerated in some cells, example images of the ER clearly portray a less densely packed peripheral region in the astrocytes than neurons (Figure 5D, top). This phenotype reiterates the Euler number findings presented in Figure 3F. ^54^ The median CV values for PO and MT were also higher in astrocytes, while the LS median CV value was higher in neurons. Utilizing the LS as another example, there was a clear difference in organelle volume per region that was distinct from the ER CV phenotype. Neurons contained a lower density of organelles throughout the somatodendritic region than astrocytes, which contained a very dense perinuclear region and a more sparsely populated peripheral region (Figure 5D, bottom). LS perinuclear clustering has been linked to differences in nutrient states, ^55^ suggesting neurons and astrocytes may contain different metabolic programming in our culture system.

The spread of organelles from the bottom to the top of the cell was variable between neurons and astrocytes, with only the ER showing no statistical differences (Figure 5B). All other organelles had a lower mode in neurons (Figure 5B, gray box), indicating the organelles were localized closer to the bottom of the cell. The PO exemplifies this phenotype well (Figure 5E). Additionally, the SD of GL, LS, and LD was greater in astrocytes, suggesting these organelles were inhabiting a wider *z* range. These findings contrasted with the *z* distribution of the nucleus (Figure S5D), which displayed only a modestly higher *z* mode in neurons. Together, our data show that neurons maintain their organelles at a lower Z level than astrocytes without a drastic difference in nucleus localization.

Our analysis also included measures of interaction site distribution (Figure 5F; Table S1); 45 interaction site distribution metrics were significantly different between cell types. However, only two were not correlated to the distribution of their constituent organelles (Figure 5F; Table S1; Figure S5E): LD-PO CV and LD-PO *z* mode. These findings indicate that LD-PO interaction volumes were more variable across *xy* regions and localized toward the top of the cell in astrocytes. The physiological significance of these findings is currently unclear.

### Neuron and astrocyte organelle signatures respond to acute stress in a cell-type- and stress-specific manner

An important aspect of cellular physiology is the ability of a cell to respond to environmental cues and maintain homeostasis. This involves the coordination and regulation of organelles, so we explored how neurons and astrocytes modify their organelle signatures in response to acute drug exposure. We selected two stress conditions commonly implicated in neurodegenerative diseases: oxidative and ER stress. ^56^ We examined their impact on organelles using the same 234 curated 3D organelle signature metrics as in Figures 1, 2, 3, 4, and 5.

Oxidative stress was induced with a 1-h 50 μM sodium arsenite exposure, sufficient to induce stress granule formation, an established cytoplasmic reorganization following arsenite treatment (Figure S6A). ^57^ ER stress was induced with a 1-h 25 nM thapsigargin exposure, which initiated the unfolded protein response, ^58^ evidenced by increased phosphorylated EIF2A (Figure S6B). Both conditions were sublethal at the 1-h imaging time point by measures of lactate dehydrogenase (Figure S6C) and 24 h after the 1-h exposure by measures of cellular metabolic activity (Figure S6D).

**Figure 6.**
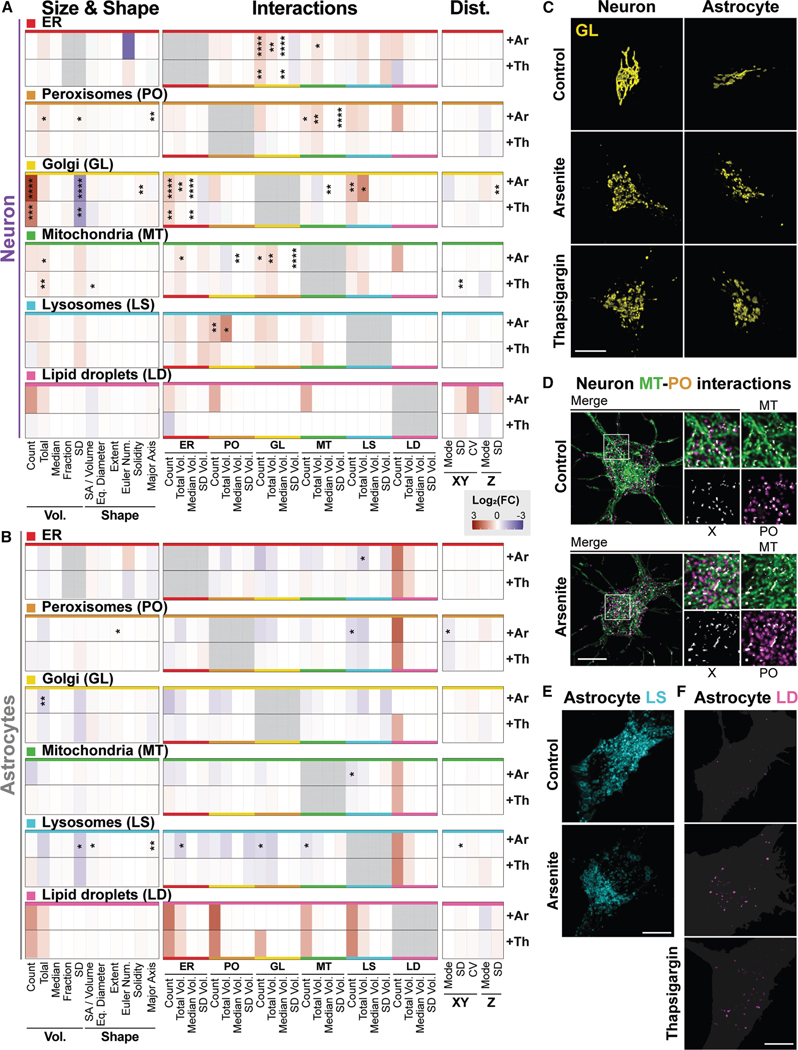
Neuron and astrocyte organelle signatures respond to acute stress in a cell-type- and stress-specific manner (A and B) Heatmaps of the log_2_ fold change (FC) in neuron (A) and astrocyte (B) 3D organelle signature metrics from baseline upon 50 μM sodium arsenite (+Ar) or 25 nM thapsigargin (+Th) exposure for 1 h; asterisks denote *q* values from the comparison of neuron and astrocyte organelle signatures (Table S3); sample size and replicate information are summarized in Figure 1D. SD, standard deviation; SA, surface area; eq., equivalent; CV, coefficient of variance. (C–F) Representative maximum intensity projections of Golgi (GL) (C), mitochondria (MT) and peroxisomes (PO) overlayed with MT-PO interaction sites (X, white) (D), lysosomes (LS) (E), and lipid droplets (LD) (F). Scale bars, 10 μm. The cell mask is represented as a gray background. Also, see Figure S6 and Table S3.

Heatmaps represent the log _2_ (fold change) of neuron and astrocyte 3D organelle signatures (Figures 6A and 6B; Table S3) from the control baselines (Figures 1, 2, 3, 4, and 5; Table S1). Both cell types responded to arsenite exposure, but only neurons showed significant organelle differences after thapsigargin treatment. Additionally, the arsenite-induced changes were more numerous than the thapsigargin-induced changes. Notably, most of these occurred in organelle interaction metrics, not organelle morphology or distribution metrics. This is likely because organelle interaction changes can be modulated rapidly by post-translational modifications, ^59^ while changes in organelle amount require transcriptional programs to induce organelle biogenesis or turnover. ^60–63^

Oxidative and ER stress share similarities in their mechanisms, ^64^ and we found evidence of this in our organelle signature dataset. Specifically, GL morphology metrics trended similarly in response to both drug exposures across cell types (Figure 6C). This shared phenotype was most strongly seen in neurons, where our data showed an increase in GL count and a reduction in the SD of volumes (Figures 6A and 6B), indicating a shift to more uniformly sized Golgi fragments. Our findings corroborate previous reports that GL fragmentation is a common response following oxidative and ER stress.^11 ,65,66^ Despite this, there are also many established mechanisms unique to arsenite or thapsigargin, especially at early time points. ^67,68^ Our dataset similarly revealed organelle distinctions between arsenite and thapsigargin responses. For example, increased MT-PO interactions were unique to arsenite-exposed neurons (Figure 6D). We found an increase in MT-PO count, total volume, and SD of volumes (Figure 6A). This phenotype was coordinated with a change in PO object morphology—increased total volume, SD of volumes, and major axis length (Figure 6A)—reminiscent of the PO clustering phenotypes in control astrocytes. Coordination of MT and PO at the interaction sites and increased PO clustering could reflect compensatory mechanisms to reduce ROS levels following arsenite damage to MT that is specific to neurons. ^69,70^

Interestingly, many of the neuron phenotypes, including increased MT-PO interactions, were not evident in astrocytes. A qualitative comparison between neuron and astrocyte stress signatures revealed opposing trends in most metrics (Figures 6A and 6B). Astrocyte-specific stress signatures were linked to reduced LS SD of volume per cell and interactions, especially in the arsenite condition, and increased LD amount and interactions trended across both drug exposures (Figures 6B, 6E, and 6F). Neither of these phenotypes was prominent in neurons, suggesting that organelle signature remodeling is strongly cell-type-specific in the face of acute oxidative and ER stress.

We also assessed differences in PO and LS dynamics following oxidative and ER stress (Figure S6E). We compared the track displacement, total distance traveled, median and standard deviation of the speeds, and tortuosity (a metric of track curvature) from 2D time-lapse images. Figure S6E shows the log_2_ (fold change) from baseline upon oxidative and ER stress. Some trends were shared across stress conditions and cell types, such as a reduction in LS speed in both cell types in response to oxidative and ER stress. In contrast, PO speed uniquely increased in neurons in response to ER stress.

### Stress-induced organelle modifications were subtle compared to cell type differences

**Figure 7.**
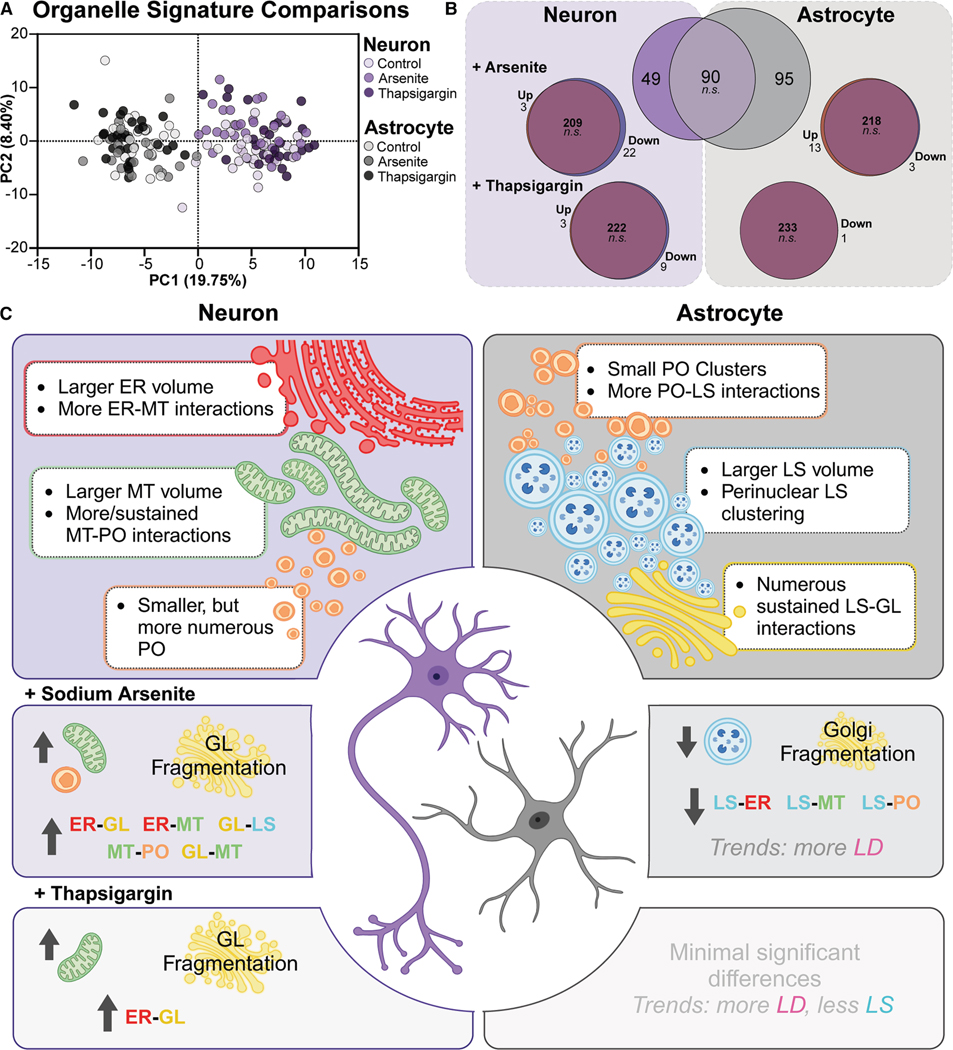
Stress-induced organelle modifications are subtle compared to cell type differences (A) Principal component (PC) scores of neurons and astrocytes under control and stress conditions discussed in Figures 2, 3, 4, 5, and 6 based on PC analysis; percentages represent variance explained by the PC. (B) Numbers of significant versus not significant (ns) differences in 3D organelle signature metrics between conditions explored in Figures 2, 3, 4, 5, and 6; statistical analyses are included in Tables S1 and S3. Sample size and replicate information are summarized in Figure 1D. (C) Data summary: organelle signatures elucidated cell-type-dependent differences between neurons and astrocytes, functionally distinct brain cell types, at baseline and in response to acute oxidative (sodium arsenite) and ER (thapsigargin) stress. Also, see Figure S7 and Tables S1 and S3.

Lastly, we compared all six drug-by-cell-type conditions. We performed a PCA with all the data discussed in Figures 1, 2, 3, 4, 5, and 6 (Figure 7A). Neurons and astrocytes were clearly separated based on PC1 irrespective of the stress condition. However, the subtle differences between stress conditions within each cell type remained discernible from the same components (Figures S7A and S7B). Analysis of the PC scores revealed shifts in the drug treatment populations along the PC2 axis. Specifically, neurons treated with arsenite and, to a lesser extent, thapsigargin had higher average PC2 scores. This contrasted with astrocytes exposed to arsenite, which had lower average PC2 scores. Additionally, neurons and astrocytes displayed opposing shifts in the average PC1 variable during thapsigargin exposure (neuron: 5.11–6.67; astrocyte: − 5.46 to − 6.43). Though subtle (Figure 7B), these differences exemplify the importance of cell type background in understanding cellular stress responses.

Hierarchical clustering revealed similar trends (Figure S7C). Again, we could separate neuron and astrocyte populations, irrespective of drug exposure. However, this analysis did not demonstrate a clear clustering of cells based on drug exposure within each cell type. It also revealed no large experiment-to-experiment batch effects in organelle signature metrics, confirming the robustness of our dataset.

## DISCUSSION

Our study presents the first systems-level characterization of organelles in live primary rodent neurons and astrocytes. Our dataset includes 173 *z* stack and 98 time-lapse confocal microscopy images of six major membrane-bound organelles—ER, LS, MT, PO, GL, and LD—from each cell. We quantified 1,418 metrics of organelle morphology, interactions, and distribution per cell from 3D images. A curated list of 234 metrics was sufficient to distinguish between cell types and elucidate nuanced alterations in organelles following acute stress (Figure 7C). Moreover, differences in organelle dynamics were discerned from 2D time-lapse images, revealing distinctions in organelle dynamics across cell types and conditions (Figures 3J and S6E). Neuron organelle signatures were higher in the MT amount and interactions with the ER and PO. Astrocyte organelle signatures contained more LS that were perinuclear. LS interactions with GL, LD, and PO were also elevated over neuron levels. An acute drug exposure further elucidated cell-type specificity, with neurons responding more robustly to stress than astrocytes.

The importance of mitochondrial function to neuron health has been robustly established.^71,72^ MT are players in axon branching and neural circuitry,^73–76^ calcium homeostasis for synaptic transmission,^77–79^ and ATP production through OXPHOS and glycolysis.^80,81^ Mechanistic studies have shown that certain mitochondrial functions, including OXPHOS, are essential to neuron survival,^82,83^ while astrocytes can withstand inhibition of OXPHOS through upregulation of compensatory mechanisms.^84,85^ MT interactions with ER and PO further this narrative. The POs are key to intracellular ROS metabolism, and coupled interactions with MT may occur more frequently in neurons to modulate ROS levels from mitochondrial OXPHOS.^69,86^ Interestingly, we noted increased MT-PO interactions during acute oxidative stress in neurons but not astrocytes. ER-MT membrane contact sites regulate mitochondrial fission, fusion, and mitophagy^6 ,87^ and maintain calcium homeostasis.^88–91^ ER-MT interactions were the most common of all 15 interaction types across cell types, but the higher amount in neurons supports the idea that MT homeostasis is important to neuronal physiology.

LS play many important roles in astrocyte functions, including involvement in gliotransmission at neuronal synapses, ^92^ exocytosis during astrogliosis, ^93^ phagocytosis of synapses, ^94^ and degradation of aberrant protein buildup in disease. ^95–98^ We observed larger LS in astrocyte images. Secretory lysosomes, large LAMP1+ vesicular structures ranging from 300 to 500 nm, ^99^ have been identified as the main exocytic vesicles involved in gliotransmission and astrogliosis. ^93,100^ Additionally, LS subcellular localization and interaction with other organelles suggest that astrocytes could have a different metabolic state than neurons in our culture system. Perinuclear clustering of LS has been linked to increased LS acidification, ^101^ autophagy, ^102^ high cholesterol availability, ^103^ and additional phenotypes.^48 ,104^ LS-GL membrane contact sites mediated by tethering complexes, such as the RAB34-RILP-FLCN complex, ^51^ are also related to the suppression of MTORC1 activity and the upregulation of autophagy. ^55,105^ Furthermore, we noted higher LS-LD and LS-PO interactions. Membrane contacts between these organelles are involved in lipid metabolism. ^106–110^ Moreover, astrocytes showed a trending increase in LD volume upon acute stress, a phenotype previously linked to protection against damaging lipid peroxidation. ^111–113^ Together, these findings suggest that astrocytes in our culture system carry out more metabolic processes related to autophagy and lipid metabolism than neurons.

We also found that some metrics were stereotyped across cell types. The distribution of organelles displayed similar trends in perinuclear to peripheral localization: GL was perinuclear; ER, LS, and MT were central; and LD and PO were peripheral. We also noted some similarities among the acute stress conditions. The largest magnitude change from baseline in all cells was related to GL fragmentation. An orthogonal machine learning (ML)-based approach in fixed induced pluripotent stem cell-derived neurons also identified alterations in GL following a 30-min, 400-μM sodium arsenite exposure. ^114^ Our data corroborate and build upon their findings by characterizing the change in GL pinpointed by their ML screening approach; our targeted metrics revealed that this alteration likely represents GL fragmentation. This phenotype is common and often reversible in many physiological and pathological conditions, ^115^ including neuron hyperexcitation, ^116^ DNA damage, ^117^ oxidative or ER stress,^11^ and cancer ^118^ ; it is also thought to be one of the earliest phenotypes in AD, suggesting that oxidative and ER stress may be involved in early disease pathogenesis. ^119^ Lastly, we noted that most of the organelle signature changes following acute stress were organelle interaction metrics. This likely reflects the modulation of membrane contact sites through post-translational modifications of tether proteins. ^59,120^ In contrast, organelle biogenesis and turnover occur on longer time scales that require transcriptional regulation. ^59,60,120^ Extended sodium arsenite and thapsigargin exposure will, therefore, likely reveal different signature phenotypes reflective of transcriptional regulation.

The organelle signatures presented here characterize commonly utilized primary cell monocultures and, therefore, can act as an organelle reference for many studies, similar to the use of omics datasets. However, *in vivo*, neurons and astrocytes directly and indirectly interact within a shared environment.^121–124^ Our findings indicate that factors derived from astrocyte media can induce subtle organelle alterations (Figure S2E). Direct and reciprocal interactions between cell types would likely impact their organelle signatures even further.^125^ Elucidating organelle signatures in co-cultures and *in vivo* models will be an exciting future direction for organelle signature analysis.

The 234 3D organelle signature metrics included above represent only a fraction of the measurements available in the full 3D dataset (Figures 1B and 1C). We demonstrated the use of additional metrics included from the larger 1,418 metric dataset in Figures 4 and S4 and hope that the full quantitative dataset will provide others with a resource to supplement their mechanistic or comparative studies in these cell types. The dataset also provides a foundation for hypothesis generation related to neuron and astrocyte physiology, cell specificity, and acute sodium arsenite and thapsigargin exposure. We encourage members of the community to explore the imaging dataset further. We supplied raw, linear unmixed, and deconvolved *z* stack and time-lapse images as well as segmentation results for *z* stack images from our BioImage archive repository. Additional analyses of segmented organelles could be used to answer specific research questions not yet answered with our quantitative methods. One such possibility would be the exploration of organelle subclasses based on size, shape, interaction types, or distribution metrics to tease out more nuanced cellular pathways and functions for future investigation. ML techniques could also be a promising approach to uncover complex or multi-organelle phenotypes that are challenging to discern through classical mathematical approaches. Likewise, the raw and linear unmixed images allow for the use of innovative unmixing and segmentation approaches or the quantitative exploration of intensity images.

Beyond this study, organelle signature analysis can be applied broadly across cell types and drug or stress conditions. Comparisons between sets of cells involved in the same tissues or diseases are a promising avenue to advance our understanding of cell specificity. The drug response variability of neurons and astrocytes shown here exemplifies the need for this type of investigation. Additionally, using the organelle signature to characterize disease-associated mutations is a powerful approach for exploring complex pathomechanisms and targets for therapeutic intervention. Disease-associated signatures then present an opportunity to test therapeutic responsiveness by monitoring system-wide markers of phenotypic improvement.

### Limitations of the study

The data included herein are specific to the cell culture models and drug conditions used. We expect that many findings can be extended to other models and conditions, but others will not. The organelle alterations detected following sodium arsenite and thapsigargin are likely to reflect only a subset of oxidative and ER stress conditions and are unlikely to recapitulate a disease setting, but rather only a stress-induced state. Additionally, the 2D monoculture system likely influences the distribution metrics collected from 3D images; previous reports indicate that the physics of 3D cultures and *in vivo* conditions impact organelle distribution substantially. ^126,127^ Nonetheless, the data collected here can serve as the foundation for hypothesis generation or support studies of the same cell types or stress conditions.

Our imaging dataset was collected from live cell cultures, not fixed cells, to ensure that static measures of organelle morphology, interactions, and distributions could be related to organelle dynamics from time-lapse images without influences from sample preparation differences. Due to physical limitations of the microscope, the imaging speeds for *z*-stack and timelapse data potentially influence our outcomes. During the collection of *z*-stack images, organelles could have moved position, leading to artifacts within the 3D data. Visual inspection indicates that the impact of this is minor or non-existent and should not influence the major outcomes presented here. Additionally, the frame rate of 5 s for time-lapse images does not allow us to detect organelle or interaction changes that could result from rapid signal transduction changes occurring in less than 5 s. Despite this, the chosen time resolution was sufficient to track organelle movement and detect differences between experimental conditions.

Our study illustrates the potential of quantitative multispectral microscopy in comparing across experimental conditions, such as cell types and acute stress exposure. However, this approach is primarily intended to screen for differences between conditions. Further experimentation using alternative approaches will be necessary to confirm our findings. For example, membrane contact sites cannot be resolved with the resolution of confocal microscopy, only changes in organelle proximity. To confirm that an increase in organelle interactions is truly due to an increase in organelle contacts, higher resolution microscopy approaches, such as electron microscopy, or other molecular methods, such as Contact-FP, ^50^ would need to be employed.

### RESOURCE AVAILABILITY

#### Lead contact

Requests for further information and resources should be directed to and will be fulfilled by the lead contact, Sarah Cohen (sarahcoh@med.unc.edu).

#### Materials availability

This study did not generate new, unique reagents.

#### Data and code availability

- The microscopy and quantitative datasets are available at BioImage archive (http://www.ebi.ac.uk/bioimage-archive). Accession numbers are listed in the key resources table.
- The code used for quantitative image analysis is available at https://github.com/SCohenLab/infer-subc.
- Any additional information required to reanalyze the data reported in this paper is available from the lead contact upon request.

## STAR★METHODS

### EXPERIMENTAL MODEL AND STUDY PARTICIPANT DETAILS

#### Primary neuron cultures

Primary cortical neurons were prepared from embryonic day 18 (E18) Sprague-Dawley rats (Charles River Laboratories, strain 001) as previously described. ^35^ Briefly, dissociated cortical neurons from embryos of both sexes were combined and plated onto tissue culture dishes coated with 1mg/mL poly-L-lysine (PLL; Sigma Aldrich). Neurons were maintained at 37^◦^C/5% CO_2_ and fed twice weekly in glial-conditioned Neurobasal media (Gibco 21130–049) supplemented with 2% B27 (Gibco 17504–044), 1% horse serum (Gibco 26050088), 2 mM Glutamax (Gibco 35050061), and 100 U/ml penicillin/streptomycin (Corning 30–002-Cl). On DIV3, 1 μM FDU (Sigma F0503) was added during feeding. The cortices of embryos, both male and female, from a single pregnant dam were combined for each experimental replicate. For live-cell imaging experiments, including multispectral microscopy, neurons were plated at 75,000 cells/well into 4-well chambered glass slides (Cellvis C4–1.5H-N); 2–4 slides were plated per experiment. The number of experimental replicates and cells per replicate are listed in Figure 1D for multispectral microscopy experiments. For fixed cell microscopy and Western blotting experiments, neurons were plated at 150,000 cells/well into 12-well tissue culture plates (Corning 3513) with or without glass coverslips (Fisher Scientific 12–545-81), respectively. For viability assays, 15,000 cells were plated per well of a 96-well plate (Thomas Scientific 1167V76). Experimental replicate information is included in the figure legends of the associated figures for non-live cell microscopy experiments.

#### Primary astrocyte cultures

Cortical astrocytes were cultured as previously described with a slightly modified protocol.^38,132^ Primary astrocytes were isolated from postnatal day 1–2 (P1-P2) Sprague-Dawley rats (Charles River Laboratories, strain 001). Cortices from both sexes were micro-dissected, combined, and enzymatically digested with papain (10 U/brain; Worthington Biochemical, LK003176L) for 25 min at 37^◦^C. The tissue was washed three times and gently aspirated to remove residual papain with astrocyte growth media (AGM), composed of DMEM high glucose (Corning, 15–013-CV0), heat-inactivated 10% FBS (VWR, 97068–085), 1X GlutaMAX (Gibco, 35050061), 1X penicillin/streptomycin (Corning, 30–002-CI), 5 μg/mL bovine insulin (Sigma-Aldrich, I6634), and 5 μg/mL N-acetyl-L-cysteine (Cayman Chemical, 20261). The tissue was triturated with three fire-polished Pasteur pipettes with progressively narrower bores in AGM and filtered through a 70-μm cell strainer (Falcon 352350). Equal volumes of the cell suspension were plated onto 75-cm ^2^ flasks (Flacon 354638) coated with 10 μg/mL of poly-D-lysine (PDL; Sigma A-003-E). Cells equivalent to the amount in one set of cortices were plated per flask. Flasks were maintained at 37^◦^C/5% CO_2_ , with a complete media exchange on DIV1 to remove debris. When astrocytes formed a confluent monolayer by DIV3, loosely adherent cells were physically dissociated by striking the flasks by hand three times in pre-warmed PBS. Cells were maintained for an additional 72 h in AGM. To encourage more stellate morphology, astrocytes were passaged into a serum-free growth medium. Cells were washed with PBS, lifted with 0.25% trypsin/EDTA (Corning, 25–053-CI), and pelleted at 125 RCF for 5 min. Cells were then washed with serum-free growth medium (NB + H), composed of phenol-free Neurobasal (Gibco, 12348017), 5 ng/mL HB-EGF (R&D Systems, 259-HE-050), 1X B27 supplement (Gibco, 17504044), 1X GlutaMAX (Gibco, 35050061), 1X penicillin/streptomycin (Corning, 30–002-CI), and then centrifuged. Counted cells were plated onto dishes freshly coated with 10 μg/mL fibronectin in PBS for 15 min at 37 ^◦^ C/5% CO _2_ and maintained in NB + H media until transfection. The cortices from three to four pups, irrespective of sex, from a single dam were combined for each experimental replicate. For live cell imaging experiments, including multispectral microscopy, astrocytes were plated in 8-well chamber glass slides (CellVis C8–1.5H-N) at 50,000 cells/well); 2–4 slides were plated per experiment. The number of experimental replicates and cells per replicate are listed in Figure 1D for multispectral microscopy experiments. For fixed cell microscopy and Western blotting experiments, astrocytes were plated at 100,000 cells/well into 24-well tissue culture plates (Corning 3526) with or without glass coverslips (Fisher Scientific 12–545-81), respectively. Experimental replicate information is included in the figure legends of the associated figures for non-live cell microscopy experiments.

All animal experiments were reviewed and approved by the Institutional Animal Care and Use Committee of the University of North Carolina at Chapel Hill (protocol 23–202.0).

### METHOD DETAILS

#### Pharmacological perturbations

Oxidative and ER stress were induced using 1-h exposures to 50 μM sodium arsenite (Sodium (meta)arsenite (Sigma-Aldrich S7400) and 25 nM thapsigargin (ThermoFischer T7458), respectively. For multispectral microscopy experiments, microscopy dishes containing live cells were maintained at 37^◦^C/5% CO_2_ in a live cell chamber on the microscope. Sodium arsenite and thapsigargin were prediluted in warm culture media and added the microscopy dish while it was in the live cell chamber. Images were collected 1 h ±10 min following the time of treatment. Drugs remained in the culture media during the imaging period. For all other experiments, the cells were treated with the indicated drugs for the indicated amount of time then prepared for downstream analysis as written below (e.g., fixed cell microscopy, cytotoxicity assays, Western blotting, etc.).

#### Multispectral imaging of organelles

##### Simultaneous organelle labeling

Primary neuron (day *in vitro*; DIV 15) or astrocyte (DIV 9) cultures were simultaneously labeled with genetically encoded and dye-based fluorescent markers for the following six organelles.

**Table:** 

Organelle	Marker	Source	Method	Concentrations for neurons	Concentrations for astrocytes
Lipid droplets (LD)	BODIPY665	Thermo Fisher Scientific, B3932	Dye	0.4 μg/mL in media	0.5 μg/mL in media
Endoplasmic reticulum (ER)	mApple-Sec61	Kind gift from Christopher Obara ^8^	Plasmid Transfection	0.7 μg	0.15 μg
Peroxisomes (PO)	mOrange2-SKL	https://www.addgene.org/54596/	Plasmid Transfection	0.07 μg	0.015 μg
Golgi (GL)	Venus-SiT	https://www.addgene.org/56336/	Plasmid Transfection	0.2 μg	0.05 μg
Mitochondria (MT)	Mito-GFP	Kind gift from Angelika Rambold ^7^	Plasmid Transfection	0.15 μg	0.015 μg
Lysosomes (LS)	Lamp1-mTurquoise	https://www.addgene.org/98828/	Plasmid Transfection	0.3 μg	0.1 μg

The labeling procedure was carried out 24 h before the start of the imaging period. For multispectral imaging of cells with all six fluorescent labels (“6-label”), the five plasmids were transfected simultaneously using Lipofectamine 2000 (Invitrogen 11–668-019) following a modified version of the manufacturer’s protocol. For one well of a 4-well chamber slide containing neurons, the indicated amount of each plasmid was added to one tube containing 50 μL of neurobasal media (Gibco 21130–049) without supplements (NB^−/−^). A 1:1 ratio of Lipofectamine 2000 to plasmid (μL:μg) was added to a second tube containing 50 μL of NB^−/−^ . Tubes were vortexed for ∼5 s, and then the plasmid dilution was pipetted into the Lipofectamine dilution. The combined solution was vortexed again and incubated at room temperature for 15 min. A 100 μL aliquot of the DNA:Lipofectamine solution was added to 500 μL of neuronal media (neurobasal + supplements as specified above) in the well of cultured neurons. The dish was rocked gently to mix. Cells were incubated in the transfection solution for 40 min at 37 ^◦^ C/5% CO_2_ . All media was removed and replaced with 500 μL of neuronal media with BODIPY665 at the above-indicated concentration. Cells were incubated at 37^◦^ C/5% CO_2_ until imaging. Astrocyte labeling in 8-well chamber slides followed the same protocol using the abovementioned astrocyte concentration and appropriate media conditions for astrocyte survival. Volumes were adjusted based on the surface area of the plate used. For single-label controls, each of the plasmids or dye was added individually; the same DNA:Lipofectamine ratio and dye concentrations were used.

It should be noted that the BODIPY665 dye, which can be used as a lipid peroxidation sensor, was selected as part of our labeling scheme due to its emission within the far-red range. Figure S1C shows the comparisons between BODIPY665 labeling and labeling with the similar BODIPY493 dye (Thermo Fisher Scientific, D3922) which lacks peroxidation sensor properties. Each dye labels the majority of the lipid droplets, but a small population are exclusively labeled by one dye or the other.

##### Multispectral imaging

Multispectral imaging was carried out as previously described. ^34^ Briefly, images were acquired on a Zeiss LSM880 laser scanning confocal microscope with a 34-channel GaAsP spectral detector and live cell incubation chamber (Carl Zeiss Microscopy). The 458 nm, 514 nm, 561 nm, and 633 nm lasers were used simultaneously to excite cells labeled with one or all of the six organelle markers. Emitted light was collected by the spectral detector in lambda mode, a linear array of 34 photomultiplier tubes (PMT), in 9.7 nm bins from 468 to 690 nm wavelengths, resulting in 26-intensity channels in the output image. A 63x/1.4 NA objective lens was used to image z stack and time-lapse images with an XYZ voxel scaling of 0.08 μm × 0.08 μm x 0.41 μm. Z-stacks were acquired at a single time point with a pixel density of 1688×1688 per slice. Each Z-slice was captured in 4.2 s. The number of slices per image was determined on a cell-to-cell basis with the goal of imaging the entire cell from top to bottom (Figure S5C). The average acquisition time per image was 117 s. The time-lapse images included a single Z-plane (pixel density of 1124×1124) imaged with Definite Focus (Carl Zeiss Microscopy) every 5 s for 5 min (60 frames). All cells (DIV16 neurons or DIV10 astrocytes) were imaged live and maintained at 37^◦^ C/5% CO_2_ for up to 2 h per dish while imaging. For both modalities, the choice to image live cells was made to ensure that the quantification of static and dynamic organelle features could be directly related without influences from differences in cell sample preparations.

##### Image processing

Spectral images were first processed into 6-channel images using the linear unmixing algorithm from ZEN Black software version 2.3 (Carl Zeiss Microscopy). Briefly, control images that were labeled with one of the six organelle markers were imaged as described above. A region of fluorescence intensity from each of the images was used as a reference spectrum for that fluorophore. All six reference spectra were utilized as inputs in the linear unmixing algorithm. The same reference spectra were used for all images within an experimental replicate. New spectra were created for different labeling conditions and cell types. The linear unmixing process resulted in a six-channel image where each channel represents the fluorescence intensity derived from each of the six fluorophores.

Linear unmixed images were then deconvolved using Huygens Essential software version 23.04 (Scientific Volume Imaging, The Netherlands, http://svi.nl). Each of the six intensity channels was deconvolved independently using the same settings across all conditions with the Workflow Processor module. Excitation and emission maxima (Ex/Em max) were estimated for each fluorophore based on the lasers utilized during imaging and the reference spectra created for linear unmixing. The following settings were used.

**Table:** 

Channel	Ex/Em max	Algorithm	Acuity	SNR	Iterations	Background
Lipid droplet	633/690 nm	Classic MLE	41.1	5.6	38	399
ER	561/593 nm	Classic MLE	−19.76	11	26	50
Golgi	561/575 nm	Classic MLE	19.33	1.5	34	340
Lysosomes	514/530 nm	Classic MLE	−49.14	6.5	26	264
Mitochondria	458/504 nm	Classic MLE	−49.83	5.4	26	52
Peroxisomes	458/477 nm	Classic MLE	81.66	5.5	53	25

Unless otherwise indicated, example images in figures include single Z-plane crops from time series or z stack images after deconvolution.

#### Cell viability

To assess the impact of experimental conditions lasting more than 1–2 h on primary cell viability (i.e., fluorescent organelle labeling methods, 24-h post-drug exposure), the CellTiter-Blue Cell Viability Assay (Promega G8080) was utilized following the manufacturer’s recommendations. In brief, primary cells were seeded in 96-well plates at the same density as imaging experiments as described above and prepared as if they were to undergo imaging. Instead of multispectral imaging at the 24-h post-labeling timepoint, CellTiter-Blue reagent was diluted 1:5 into pre-warmed culture media and incubated on the cells for 4 h at 37^◦^ C. Fluorescence (560Ex/590Em) was measured using a FLUstar Omega Plate Reader (GMB LabTech).

#### Cytotoxicity

To assess the level of cytotoxicity from experimental conditions that lasted 2 h or less (e.g., 1 h drug exposures), CytoTox 96 Non-Radioactive Cytotoxicity Assay (Promega G1780) was utilized. The manufacturer’s protocol was followed. Briefly, three replicates of 50 μL of media from cultured cells were aliquoted into wells of a 96-well plate for all conditions tested. Media from unmanipulated cells of the same age were included as a negative control within each experiment. An equal volume of CytoTox 96 reagent was added and incubated in the dark at room temperature for 30 min. Stop solution was added to each well and bubbles were removed before reading the absorbance at 490nm.

#### Western blotting

Cells were lysed in ice-cold RIPA buffer (50mM Tris pH 8.0, 150mM NaCl, 1%NP-40, 5mM EDTA, 0.5% sodium deoxycholate, 0.1%SDS) containing additional inhibitors (2.5 mM β-glycerolphosphate (Sigma G9422) 2.5 mM NaF (Sigma S7920), 10 mM nicotinamide (Sigma 72340), 1 mM sodium orthovanadate (Alfa Aesar AA8110414), 1 mM phenylmethylsulfonyl fluoride (PMSF; Fisher Scientific 52–332), 1 μM Trichostatin A (Sigma T8552), 1X Protease Inhibitor Cocktail (Promega G6521), 2 μM okadaic acid (Sigma O9381). Protein samples were denatured in a mix of 6X sample buffer (Fisher Scientific 50–591-186) with dithiothreitol (DTT) and boiled at 98^◦^C for 10 min before running on 12% Criterion TGX Precast Protein Gels (Bio-Rad 5671044). Samples were transferred to 0.2 μm nitrocellulose membrane (Bio-Rad 162–0112) by wet transfer. Total protein amounts were assessed using PonceauS (Research Products International Corp P56200) protein stain and destained following manufacturer instructions. Membranes were blocked in 2% nonfat milk (Lab Scientific bioKEMIX 978–907-4243) in 1X TBS (Sigma T5912) for 30 min at room temperature while rocking. Primary antibodies were diluted into 2% milk and incubated overnight at 4^◦^ C while rocking. Membranes were washed three times in 1X TBST (1X TBS with 0.1% Tween 20; Sigma P9416), then incubated for one hour at room temperature while rocking in secondary antibody diluted in 2% milk. The membranes were washed again in the same way and then switched into 1X TBS for a final wash. Chemiluminescent or near-infrared immunofluorescence imaging was carried out using an ImageQuant LAS4000 machine or a Li-COR Odyssey CLx Infrared Imaging System (Li-COR Biosciences; Lincoln, NE), respectively. Phosphorylated eIF2α, VAPB, and TOM20 was detected with 1:1000 rabbit anti-phospho-eIF2 (Cell Signaling CS3398), 1:5000 rabbit anti-VAPB (Proteintech 14477–1-AP), 1:10,000 rabbit anti-TOM20 (Proteintech 11802–1-AP) primary antibodies, respectively, and 1:2000 goat anti-rabbit-HRP (Invitrogen 32460) secondary antibodies; GAPDH was detected with 1:500 chicken anti-GAPDH (Sigma AB2303 primary and 1:10,000 goat anti-chicken IRDye 800CW (LI-COR 926–32211) secondary antibodies; PMP70 and PTPIP51 were detected with 1:1000 mouse anti-PMP70 (Sigma SAB4200181) and 1:1000 mouse anti-PTPIP51 (Proteintech 68582–1-Ig) primary antibodies, respectively, and 1:10,000 goat anti-mouse (Invitrogen 31432) secondary antibodies.

#### Stress granule imaging

Primary neurons or astrocytes cultured on glass coverslips at the same plating density as was used for live cell imaging experiments were fixed for 10 min at 37^◦^ C/5% CO2 in a solution containing 2% paraformaldehyde (PFA; Electron Microscopy Sciences 15713) and 0.05% glutaraldehyde in PHEM buffer (EMS, 11162) prewarmed to 37^◦^ C. Coverslips were rinsed three times in PBS (Corning 21–040) and permeabilized in 0.2% Triton X-100 (Sigma X100) for 8 min at room temperature. Coverslips were rinsed again in PBS, incubated in 2% milk for 1 h at room temperature, and then incubated in primary antibody solution overnight at 4^◦^ C in a humid chamber. Two markers of stress granules, G3BP1 and TIA1, were used for detection: 1:3000 rabbit anti-G3BP1 (Protein Tech T3057–2-AP, lot 000–5872) and 1:250 goat anti-TIA1 (Santa Cruz SC-1751, lot E1314). Coverslips were rinsed again in PBS, incubated in secondary antibody diluted in 2% milk for 1 h at room temperature in the dark, rinsed again, and mounted to slides using SlowFade Mounting Medium with DAPI (ThermoFisher S36973) and sealant (Biotium 23005). The following secondary antibodies were used serially: staining round 1 used 1:500 donkey anti-goat-AF488 (Invitrogen A11055, lot 1627966) followed by 1:500 goat anti-rabbit-AF568 (Invitrogen A11011, lot 2088069). After curing for at least 24 h at room temperature, slides were imaged. Images were collected with a Zeiss LSM800 point scanning confocal microscope (Carl Zeiss Microscopy) using a 63X/1.4 NA oil immersion objective with 405 nm, 488 nm, and 561 nm lasers.

#### Summary diagrams

The summary diagrams in Figures 1A, 6C, and S4A were created in Biorender:

Figure 1A: Created in BioRender. Cohen, S. (2024) https://BioRender.com/t11q270.

Figure 6C: Created in BioRender. Cohen, S. (2024) https://BioRender.com/b84o183.

Figure S4A: Created in BioRender. Cohen, S. (2024) https://BioRender.com/z12e684.

#### Graphing and data visualization

Boxplots, heatmaps, and dendrograms were created using R coding language. ^133^ Boxplots and heatmaps were created with ggplot2. ^129^ Boxplots display the median, range, and interquartile range. Individual data points represent per-cell values. Dendrograms were generated with Pretty Heatmaps as described above. ^128^

PC scores scatterplots, volcano plots, bar charts, dot plots, and pie charts were created in GraphPad Prism 10.2.3 Windows, GraphPad Software, Boston, Massachusetts USA, www.graphpad.com.

#### Data storage and availability

All imaging and quantitative data is available at BioImage Archive accession number S-BIAD1445. ^134^

### QUANTIFICATION AND STATISTICAL ANALYSIS

#### 3D organelle signature analysis

##### 3D image segmentation

Instance segmentations were created from independent intensity channels in the deconvolved z stack images. Methods for segmentation are included and described in detail in our Python-based segmentation and analysis package, infer-subc version 1.0 (https://github.com/ndcn/infer-subc). They combine previously published segmentation methods optimized for organelles from the Allen Cell Segmenter “Classic Image Segmentation Workflow” ^135^ and methods from the scikit-image Python package. ^136^ Semantic segmentation was performed first, followed by instance segmentation based on connectivity. Specifically, organelle objects found in the semantic segmentation were considered separate objects if they did not neighbor another object in any direction (X, Y, Z, or diagonally). Because of this, organelles within one voxel’s distance are considered the same object.

The cell mask and nucleus were inferred from a combination of all six organelle labels using similar methods. The nucleus was considered the absence of organelle fluorescence intensity in the central area of the soma or cell body. Astrocyte images usually contain multiple cells per field of view; a single cell with optimal labeling intensities was selected from each image for quantitative analysis. Neuron images only contained the somatodendritic region of a single neuron each. The ER, cell mask, and nucleus mask underwent semantic segmentation to produce a single object per image. This decision was made *a priori* based on the knowledge that each cell will only contain one ER, cell area, and nucleus.

The same segmentation settings were applied to all images. Qualitative checks were performed to ensure the accuracy of each segmentation output. If errors were found, the segmentations were manually edited. In neuron images, 20.55% of the segmentations were manually edited; in astrocyte images, 35.86% were manually edited. Edits to the cell mask and nucleus comprise 69.53% of the manually edited segmentations.

##### 3D organelle quantification (1418 metrics)

Quantitative analysis of neuron and astrocyte organelle signatures is included and described in detail in infer-subc (https://github.com/ndcn/infer-subc/tree/main/notebooks). After segmentation, organelle objects and interactions between organelles, defined as the region of overlap between two organelle objects, were quantified for features of morphology (e.g., amount, size, and shape) and distribution. The morphology of the cell mask and the morphology and distribution of the nucleus mask were also quantified. The analyses were conducted from the entire cell volume included in the field of view in each image. For neurons, this includes the soma, proximal dendrites and axon initial segment; proximal dendrites and axons—together the proximal neurites—were not distinguished from one another. All per-object measurements were summarized per cell, resulting in 1418 organelle signature metrics.

The full list of organelle signature metrics, metric definitions, and per-object quantification is included in our BioImage Archive data repository.

##### Organelle interactions

Organelle interactions were assessed between pairwise combinations of the six organelles. Organelle interaction sites were defined as regions of overlap between individual organelle objects. Specifically, after organelle segmentation from 3D multispectral images, regions of overlap between each pairwise combination of organelles (e.g., ER and LS is a single pair) were extracted and measured for features of morphology and subcellular distribution (see descriptions of these measurements below).

##### Morphology measurements

Measurements of object morphology were collected to quantify the amount, size, and shape of each organelle and interaction site. We used the scikit-image skimage.measure.regionprops_table function ^136^ including the following measurements for each object: label, centroid, bounding box, area (i.e., volume), equivalent diameter, extent, Euler number, solidity, and major axis length. The minimum intensity, maximum intensity, and mean intensity were also included for each channel in the cell mask quantification to allow for assessment of the fluorescence intensity of the organelle markers. Definitions of each property are included in sci-kitimage documentation here: https://scikit-image.org/docs/stable/api/skimage.measure.html#skimage.measure.regionprops. The analyses are further summarized and were run utilizing infer_subc.utils.stats.get_org_morphology_3D and infer_subc.utils.stats.get_region_morphology_3D functions. All measurements were collected in “real-world” units (e.g., μm, nm), not voxel units.

##### Distribution measurements

Distribution measurements were taken to assess the spread of organelles and interaction sites from the nucleus to the cell membrane (XY) and from the bottom to the top of the cell (Z). Quantification of the XY distribution is based on the ideas presented in MeasureObjectIntensityDistribution module included in CellProfiler. ^137^ Briefly, a sum projection of each cell mask was created. Then, concentric rings proportional to the nucleus and cell shape were drawn, beginning at the edge of the nucleus mask and ending at the edge of the cell mask for each cell (Figure S5A, bottom). Each ring was utilized as a separate subcellular region from which we measured the volume of each organelle and interaction site from all Z planes. The central most ring included the nucleus and volumes above and below it, and the most peripheral ring included all the neurites in neurons or projections in astrocytes. To quantify the Z distribution, the bottom and top of the cell were defined by the lowest and highest Z planes that contained a portion of the cell mask, respectively. The Z planes were separated into ten approximately equal regions from which organelle volume was measured.

Each XY and Z region contained a different cell mask volume depending on the cell’s shape (Figures S5B and S5C). To control for this and differences in organelle amount per cell, the organelle, interaction site, and nucleus volume values were normalized to total object volume and region size. We represented the normalized volumes as histograms (Figures 5A, 5B and S5D). Histogram summary statistics were then used to summarize the spread of the organelles across the set of XY and Z regions more succinctly. Two histogram summary statistics, mode and standard deviation (SD), were included in the curated organelle signature analysis to succinctly summarize the XY and Z distributions (Figures 5A and 5B, gray boxes; Table S1). To assess the variance in volume within each XY region, we also calculated the coefficient of variance per organelle and interaction site volume within each XY region. Beginning at the center of the nucleus, radial lines were created to divide each XY region into eight equal fractions (Figure S5A, bottom panel, red dashed lines). CV of the normalized volumes between each of the eight sections was calculated for each organelle and interaction site, and the median value per cell was included in the statistical analysis. The median CV per cell was included in the curated organelle signature analysis (Figure 5C).

These analyses were run using infer_subc.utils.stats.get_XY_distribution and infer_subc.utils.stats.get_Z_distribution functions.

##### Batch processing quantification

Quantification for all images in each experiment was batch-processed using the infer_subc.utils.make_all_metrics_tables function. All experimental replicates were combined, and the data was summarized per cell using the infer_subc.utils.batch_summary_stats function. This produced the 1418 organelle signature metrics. Quantification for each experiment and the summary statistics per cell for the entire dataset are available in our repository on BioImage Archive.

##### Organelle signature variable curation (234 metrics)

To reduce redundancy and improve statistical power for the statistical comparisons included here, the 1418 organelle signature metrics were narrowed down to 234 metrics that were used to screen major differences between conditions in this study. The variables were chosen *a priori* and without bias toward known cell-type- or stress-specific outcomes to increase the potential of seeing differences in organelles irrespective of the biological comparison being made. The values summarized per cell are described in Table S4.

##### Outliers

Outlier cells were considered based on the values of all 234 organelle signature metrics, not each metric individually. No outliers were removed from the dataset prior to statistical analysis.

##### Principal Component Analysis

Principle Component Analysis (PCA) was carried out using the GraphPad Prism 10.2.3 Windows, GraphPad Software, Boston, Massachusetts USA, www.graphpad.com. The curated list of 234 organelle signature metrics was first preprocessed by removing variables with a standard deviation of 0 and then standardized so that each variable had a mean of zero and a standard deviation of 1. Principle components (PCs) were selected using parallel analysis (1000 Monte Carol simulations; selection based on eigenvalues greater than the 95% percentile in simulation; random seed auto-selected). The list of removed variables from each comparison is included in Table S5.

##### Pairwise comparisons

Pairwise comparisons between cell types and drug exposure conditions were made using a Mann-Whitney U test in GraphPad Prism 10.2.3 Windows, GraphPad Software, Boston, Massachusetts USA, www.graphpad.com. Significance was determined using the two-state linear step-up procedure of Benjamini, Kreiger, and Yekutieli with a False Discovery Rate (FDR) of 10% (q = 0.1). All 234 organelle signature metrics were initially included in the analysis. However, metrics were removed if either comparison group contained one or fewer data points (e.g., only one cell or no cells have LD, so there were insufficient volume data points). Significance is denoted as follows: q > 0.1 (no significance), q ≤ 0.1 (*), q ≤ 0.05 (**), q ≤ 0.01 (***), and q ≤ 0.001 (****). The q-value and mean rank difference from this analysis, along with the number of cells (N), mean, median, minimum, maximum, standard deviation (SD), and percent coefficient of variance (%CV) for each experimental condition are summarized for neuron versus astrocyte and control versus drug exposure conditions in Tables S1 and S3, respectively.

Metrics outside of the curated analysis were analyzed independently. Specifically, the percent PO in LS-PO interactions (Figure 3D) and the cell morphology metrics (Figure S1F) utilized Mann-Whitney U tests while organelle total surface area comparisons between organelles (Figure S4C) utilized Kruskal-Wallis non-parametric One-Way ANOVA test with Dunn’s multiple comparison test; all analyses were carried out in GraphPad Prism 10.2.3 Windows, GraphPad Software, Boston, Massachusetts USA, www.graphpad. com. The cell morphology measurements included in Figure S1F include cell_axis_major_length, cell_equivalent_diameter, cell_euler_number, cell_extent, cell_SA_to_volume_ratio, cell_solidity, cell_surface_area, cell_volume, nuc_area_fraction, nuc_axis_major_length, nuc_equivalent_diameter, nuc_euler_number, nuc_extent, nuc_SA_to_volume_ratio, nuc_solidity, nuc_surface_area, and nuc_volume. P-value significance is denoted as follows: *p* > 0.05 (ns), *p* ≤ 0.05 (*), *p* ≤ 0.01 (**), *p* ≤ 0.001 (***), and *p* ≤ 0.0001 (****).

##### Correlation

The correlation of organelle signature metrics was based on Spearman’s correlation (r) and calculated along with *p*-values for significance in GraphPad Prism 10.2.3 Windows, GraphPad Software, Boston, Massachusetts USA, www.graphpad.com. P-value notation is summarized above. Spearman’s r and *p*-values for all correlation analyses are included in Table S2.

##### Hierarchical clustering

Hierarchical clustering was performed in R using the Pretty Heatmaps package version 1.0.12. ^128^ The list of organelle signature metrics processed for PCA (i.e., metrics with SD of 0 removed and data normalized) was used in this analysis. Cells were clustered using the ward.D method and Euclidian distance measurement.

#### Quantification of organelle dynamics from 2D timelapse images

The dynamics of LS and PO were quantified from 2D time-lapse datasets using TrackMate and Celltrackcolab. ^130,131^ Cell tracking was conducted through the Fiji plug-in, TrackMate (version 7.14.0). LS, PO, and LD were tracked in 95 cells (64 astrocytes and 31 neurons). TrackMate Batcher was used to apply the same parameters across each cell. Tracking outputs were later manually inspected after batch processing to ensure accuracy; several astrocyte images were excluded from the analysis as they had excessive cell movement, causing them to drift out of the cell mask generated using frame one. The final dataset included 31 neurons and 30 astrocytes evenly distributed across the treatment and control conditions. The XML files from TrackMate were imported into CellTracksColab, ^131^ a program for visualizing and analyzing tracking data. CellTracksColab version v1.0.1 was used following the recommended data structure and analysis steps. Metrics of organelle dynamics listed below were included in Figures 2 and S6; the full analysis dataset is included in the Bioimage Archive data repository. Significance was determined using the Bonferroni-corrected statistical outcomes from the CellTracksColab analysis.

Measurements included from the CellTracksColab analysis.

**Table:** 

Metric	Definition
Track displacement	The length of a straight line connecting the location of the tracked object in the first frame to the location of the tracked object in the last frame.
Total distance traveled	The sum of the distance traveled by the tracked object throughout the time-lapse image.
Median speed (rolling)	The median speed of the tracked object was calculated for rolling windows of 10 frames across all tracks. The rolling window makes comparisons of the speed across tracks of different lengths more consistent.
Standard deviation of speed (rolling)	The standard deviation of speed of the tracked object was calculated for rolling windows of 10 frames across all tracks. The rolling window makes comparisons of the speed across tracks of different lengths more consistent.
Tortuosity (rolling)	A measure of the curvature and complexity of the path taken by the tracked object; this value was calculated for rolling windows of 10 frames across all tracks. The rolling window makes comparisons of the speed across tracks of different lengths more consistent. A value of 1 indicates a straight path between start and end. More information about tortuosity can be found at https://github.com/CellMigrationLab/CellTracksColab.

#### Cell viability analysis

Individual data points were background subtracted using the average fluorescence value from the “dead” negative control (70% EtOH exposure for 30 min) wells and normalized using the average fluorescence from the positive control (cells only) wells. Statistical comparison of conditions to the positive control (unmanipulated cells) was done using a repeat measures one-way ANOVA with a Geisser-Greenhouse correction and Dunnett’s test for multiple comparisons in GraphPad Prism 10.2.3 Windows, GraphPad Software, Boston, Massachusetts USA, www.graphpad.com. Neuron and astrocyte data were compared separately and consisted of two biological replicates with up to three technical replicates each.

#### Cytotoxicity analysis

Percent cytotoxicity was calculated by dividing the average of the experimental replicates by the average of the “dead” cell control and multiplying by 100. Statistical comparison of conditions to the vehicle control was done using a one-way ANOVA with Dunnett’s test for multiple comparisons with a single pooled variance in GraphPad Prism 10.2.3 Windows, GraphPad Software, Boston, Massachusetts USA, www.graphpad.com. The drug exposure conditions included in these assays were not labeled with the fluorophores used for imaging.

#### Western Blot analysis

Quantification was carried out in Li-COR ImageStudio Software version 5.2. Boxes were drawn around antibody bands of the correct molecular weight and the band intensity with the relative background was collected in the ImageStudio software. The intensity signal of each band was normalized using the normalized GAPDH signal from the same lane.

#### Stress granule analysis

The percentage of cells that contained stress granules was counted manually. Images were processed for visualization in ImageJ Fiji148. Example images are maximum intensity projections.

#### Neuron cell mask skeleton analysis

ImageJ/Fiji was used to analyze neuron branching differences between astrocyte-conditioned and astrocyte-naı¨ve neurons. The script included in Data S1 was run on all neurons included in the multispectral dataset. Briefly, binary cell masks generated during segmentation for organelle signature analysis were flattened into 2D maximum intensity projections and then skeletonized. Skeleton analysis was carried out, and select metrics were compared by Student’s t-test and plotted in GraphPad Prism 10.2.3 Windows, GraphPad Software, Boston, Massachusetts USA, www.graphpad.com.

## Supplementary Material

1

2

3

4

5

6

7

SUPPLEMENTAL INFORMATION

Supplemental information can be found online at https://doi.org/10.1016/j.celrep.2025.116280.

## Figures and Tables

**Table:** KEY RESOURCES TABLE

REAGENT or RESOURCE	SOURCE	IDENTIFIER
Antibodies		
Phospho-eIF2α (Ser51) (D9G8) XP^®^ Rabbit mAb	Cell Signaling Technology	RRID:AB_2096481
Goat anti-Rabbit IgG (H + L) Secondary Antibody, HRP	ThermoFischer Scientific	RRID:AB_1185567
Anti-GAPDH Antibody	Sigma-Alderich	RRID:AB_10615768
IRDye 800CW Goat anti-Rabbit IgG	Li-COR Biosciences	RRID:AB_621843
VAPB Polyclonal antibody	ProteinTech	RRID:AB_2288297
TOM20 Polyclonal antibody	ProteinTech	RRID:AB_2207530
Anti-PMP70 antibody, Mouse monoclonal	Millipore-Sigma	RRID:AB_10639362
PTPIP51 Monoclonal antibody	ProteinTech	RRID:AB_3085282
G3BP1 Polyclonal antibody	ProteinTech	RRID:AB_2232034
TIA-1 Antibody (C-20)	Santa Cruz Biotechnology	RRID:AB_2201433
Invitrogen Donkey anti-Goat IgG (H + L) Cross-Adsorbed Secondary Antibody, Alexa Fluor™ 488	ThermoFischer Scientific	RRID:AB_2534102
Invitrogen Goat anti-Rabbit IgG (H + L) Cross-Adsorbed Secondary Antibody, Alexa Fluor™ 568	ThermoFischer Scientific	RRID:AB_143157
Chemicals, peptides, and recombinant proteins		
Gibco™ Neurobasal™ Medium	Fischer Scientific	Cat#21130–049
Gibco™ B-27™ Supplement (50X), serum free	ThermoFischer Scientific	Cat#17504044
Poly-L-lysine solution	Sigma-Aldrich	Cat#P8920
Gibco™ Horse Serum, heat inactivated, New Zealand origin	ThermoFischer Scientific	Cat#26050088
Gibco™ GlutaMAX™ Supplement	ThermoFischer Scientific	Cat#35050061
Corning™ Penicillin-Streptomycin Solution	Fischer Scientific	Cat#MT30001CI
5-Fluoro-2′-deoxyuridine	Sigma-Aldrich	Cat#F0503
Papain	Worthington Biochemical Company	Cat#LK003176L
DMEM, Corning^®^ 500 mL DMEM (Dulbeccos Modification of Eagles Medium) [+] 4.5 g/L glucose, sodium pyruvate [-] L-glutamine	Avantor	Cat#15–013-CV
Premium Grade Fetal Bovine Serum (FBS)	Avantor	Cat#97068–085
Insulin from bovine pancreas	Sigma-Aldrich	Cat#I6634
N-acetyl-L-Cysteine	Cayman Chemical	Cat#20261
Poly-ᴅ-Lysine Hydrobromide	Millipore-Sigma	Cat#A-003-M
Corning™ 0.25% Trypsin, 0.1% EDTA in HBSS w/o Calcium, Magnesium and Sodium Bicarbonate	Fischer Scientific	Cat#MT25053CI
Gibco™ Neurobasal™ Medium, minus phenol red	ThermoFischer Scientific	Cat#12348017
R&D Systems™ Recombinant Human HB-EGF Protein	Fischer Scientific	Cat#259HE050
Sodium (meta)arsenite	Sigma-Aldrich	Cat#S7400
Invitrogen™ Thapsigargin	ThermoFischer Scientific	Cat#T7458
Invitrogen ™ BODIPY ™ 665/676 (Lipid Peroxidation Sensor)	ThermoFischer Scientific	Cat#B3932
Invitrogen™ Lipofectamine™ 2000 Transfection Reagent	Fischer Scientific	Cat#11–668-019
Tris Buffer, 1.0 M, pH 8.0, Molecular Biology Grade	Millipore-Sigma	Cat#648314
Sodium chloride	Millipore-Sigma	Cat#S9888
NP-40 Surfact-Amps™ Detergent Solution	ThermoFischer Scientific	Cat#85124
UltraPure™ 0.5M EDTA, pH 8.0	ThemoFischer Scientific	Cat#15575020
Thermo Scientific™ Sodium Deoxycholate Detergent	ThemoFischer Scientific	Cat#89904
Sodium dodecyl sulfate	Millipore-Sigma	Cat#436143
β-Glycerophosphate disodium salt hydrate	Millipore-Sigma	Cat#G9422
Sodium fluoride	Millipore-Sigma	Cat#S7920
Nicotinamide	Millipore-Sigma	Cat#72340
Sodium orthovanadate, 99.9% (metals basis), Thermo Scientific Chemicals	Fischer Scientific	Cat#AA8110414
MilliporeSigma™ Calbiochem™ Phenylmethylsulfonyl Fluoride	Fischer Scientific	Cat#52–332
Trichostatin A	Millipore-Sigma	Cat#T8552
Protease Inhibitor Cocktail	Promega	Cat#G6521
Okadaic acid	Millipore-Sigma	Cat#O9381
New England Biolabs, Inc. Gel Loading Dye Purple (6X) – 4 mL	Fischer Scientific	Cat#50–591-186
DTT (dithiothreitol)	ThemoFischer Scientific	Cat#R0861
Tris-Buffered Saline	Millipore-Sigma	Cat#T5912
TWEEN^®^ 20	Millipore-Sigma	Cat#P9416
Paraformaldehyde 20% Aqueous Solution EM Grade	Electron Microscopy Sciences	Cat#15713
PHEM Buffer, 0.1M	Electron Microscopy Sciences	Cat#11162
Glutaraldehyde Solution	Millipore-Sigma	Cat#354400
Corning^®^ Phosphate-Buffered Saline, 1X without calcium and magnesium, pH 7.4 ± 0.1	Corning	Cat#21–040
Triton™ X-100	Millipore-Sigma	Cat#X100
Invitrogen™ SlowFade™ Diamond Antifade Mountant with DAPI	ThemoFischer Scientific	Cat#S36973
CoverGrip™ Coverslip Sealant	Biotium	Cat#23005
Critical commercial assays		
CellTiter-Blue^®^ Cell Viability Assay	Promega	Cat#G8080
CytoTox 96^®^ Non-Radioactive Cytotoxicity Assay	Promega	Cat#G1780
Deposited data		
BioImage Archive	This paper	S-BIAD1445
GitHub	This paper	https://github.com/SCohenLab/infer-subc
Experimental models: Organisms/strains		
Sprague-Dawley Rats	Charles River	RRID:RGD_734476
Recombinant DNA		
mApple-Sec61	Christopher Obara	Nixon-Abell et al.^8^
mOrange2-Peroxisomes-2	Addgene	Cat#54596
mVenus-SiT-N-15	Addgene	Cat#56336
Mito-GFP	Angelika Rambold	Rambold et al.^7^
Lamp1-mTurquoise2	Addgene	Cat#98828
Software and algorithms		
ZEN Black	Carl Zeiss Microscopy	Version 2.3
Huygens Essential	Scientific Volume Imaging	Version 23.04
infer-subc	This paper	Version 1.0
Prism	GraphPad	Version 10.2.3
Pretty Heatmaps	Raivo Kolde ^128^	Version 1.0.12
ImageStudio	Li-COR Biosciences	Version 5.2
ggplot2	Wickham et al. ^129^	Version 3.5.2
TrackMate	Ershov et al. ^130^	Version 7.14.0
CellTracksColab	Gómez-de-Mariscal et al.^131^	Version 1.0.1

## Highlights

- Neuron and astrocyte “organelle signatures” are distinct and reflect cell functions
- Neuron signatures exhibit mitochondrial, ER, and peroxisomal phenotypes
- Astrocyte signatures contain lysosomal, Golgi, and lipid droplet phenotypes
- Organelle signatures remodel in a cell-type- and stress-specific manner
