# Supplementary material for: Neurons and astrocytes have distinct organelle signatures and responses to stress
Source: Cell Rep. Author manuscript; Available in PMC 2025 Nov 20. (PMC12631801; doi:10.1016/j.celrep.2025.116280)
Supplement: 1 [file NIHMS2113415-supplement-1.pdf]

Cell Reports, Volume *44*

Supplemental information

Neurons and astrocytes have distinct organelle signatures and responses to stress

Shannon N. Rhoads, Weizhen Dong, Chih-Hsuan Hsu, Ngudiankama R. Mfulama, Ridhi Yarlagadda, Joey V. Ragusa, Michael Ye, Andy Henrie, Maria Clara Zanellati, Graham H. Diering, Todd J. Cohen, and Sarah Cohen

Supplemental Figures:

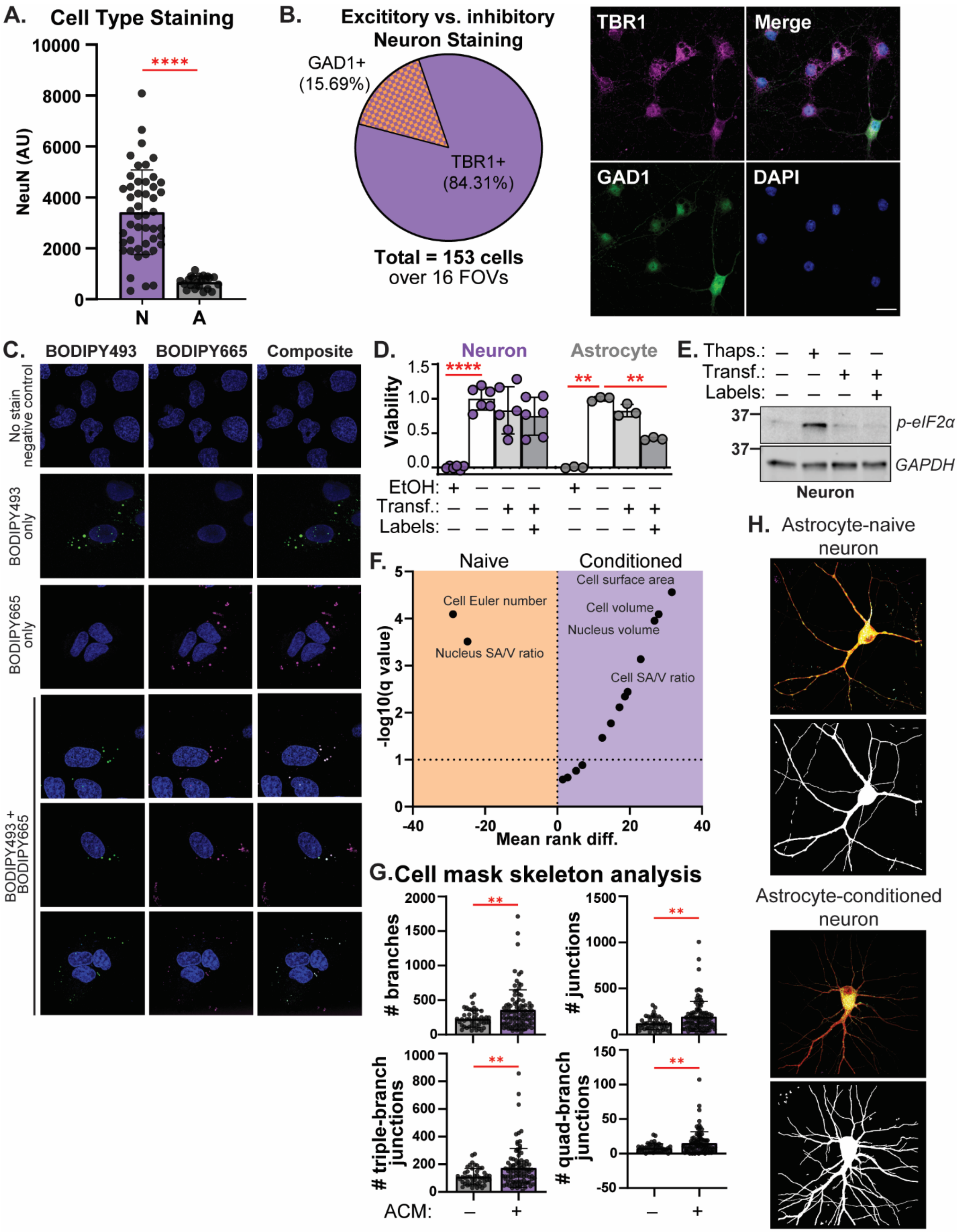

Figure S1. Primary cell culture and multispectral imaging controls. A. Quantification of cell-

type-specific immunostaining of fixed neuron (N) and astrocyte (A) monocultures used for multispectral imaging (n=3); each datapoint represents the NeuN intensity per cell. B. Representative sum intensity projections (right) and quantification (left) of the portion of excitatory (TBR1+) and inhibitory (GAD1+) neurons from the neuron monocultures used for multispectral imaging (n=2). DAPI stain indicates localization of the nuclei. C. Comparison between fluorescent BODIPY lipid dyes; live cells labeled with DAPI (blue) nuclear stain, the BODIPY665 peroxidation sensor that was used as part of the multispectral imaging suite, and/or BODIPY493. D. Cell viability following the addition of fluorescent organelle markers ("labels") used during multispectral imaging experiments or transfection reagent alone ("Transf."). Bars represent the mean ± SD of the normalized CellTiter-Blue intensity measurement; individual data points represent experimental replicates across all biological replicates (n=2). E. Representative western blot results assessing activation of the unfolded protein response (UPR) via phosphorylated eIF2α levels following multispectral imaging preparation in neurons (n=3). F. Volcano plot comparing cell morphology metrics from the full 3D dataset between astrocyte-naïve and astrocyte-conditioned neurons; significance was determined by 10% false discovery rate. G. Quantitative features of neuron cell masks after skeletonization, including the number of branches, junctions (connections between branches), triple-branch junctions (junctions between 3 branches), and quadruple-branch junctions (junctions between 4 branches), were compared between astrocyte-naïve and astrocyte-conditioned neurons. F-G. Astrocyte-conditioned neuron sample sizes and replicate information are summarized in Figure 1D; astrocyte-naïve neuron data includes cells from 3 biological replicates. H. Representative maximum intensity projections of multispectral intensity images and cell masks of astrocyte-naïve and astrocyte-conditioned neurons used to quantify neuron shape in panels F-G.

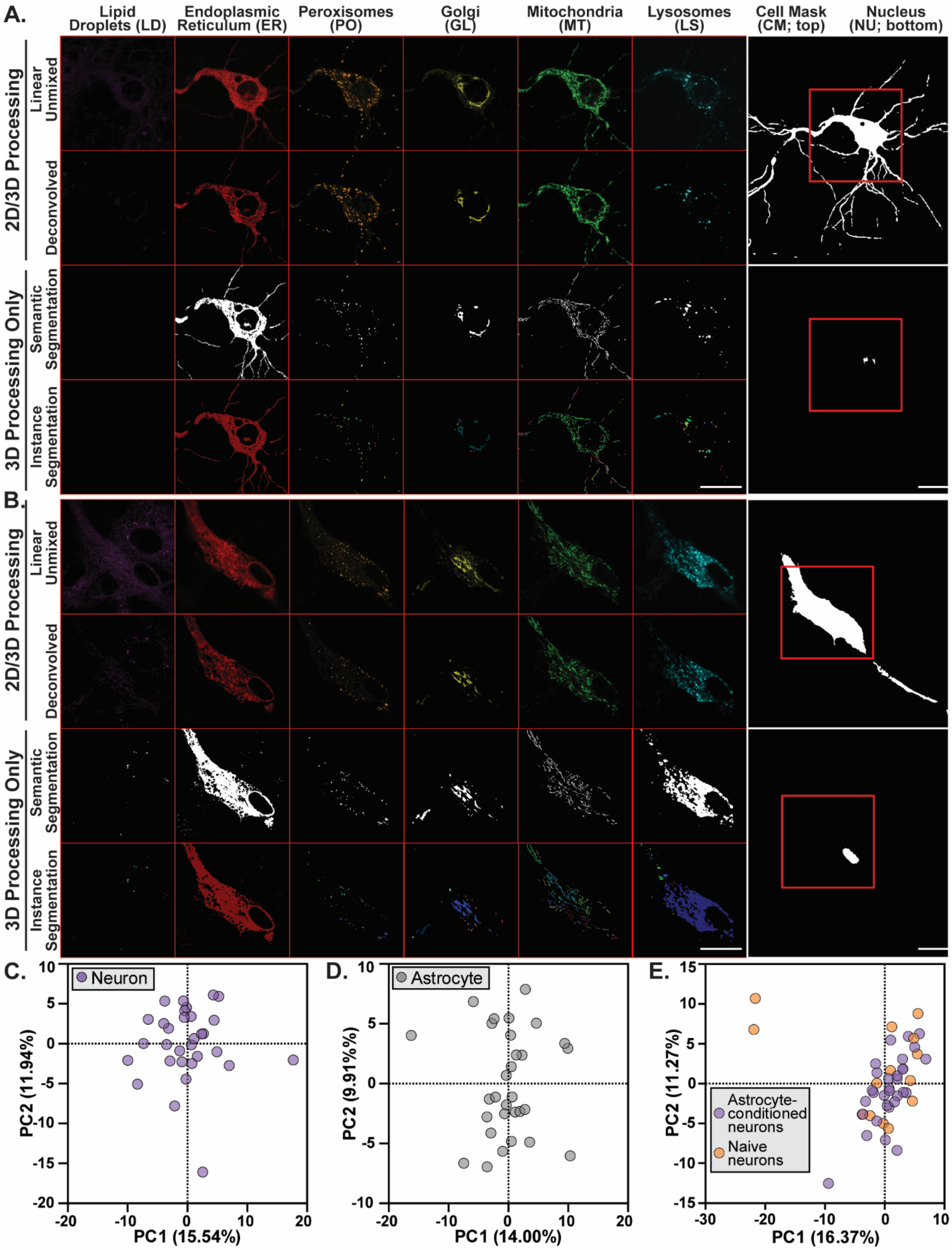

Figure S2. Multispectral image processing and segmentation outputs, and independent organelle signature analysis of neurons and astrocytes, related to Figure 2. A-B. Representative linear

unmixing, deconvolution, and segmentation outputs for the example neuron (A) and astrocyte (B) included in Figure 2A-B; scale bars represent 20 μm. C-E. Principal component (PC) scores from PC analysis of control neurons (C), control astrocytes (D), and astrocyte-naïve versus control (astrocyte-exposed) neurons (E) 3D organelle signatures; percentages represent percent variance explained by the respective PC. C-D. Control neuron and astrocyte sample sizes and replicate information are summarized in Figure 1D. E. Astrocyte-naïve neuron data includes 14 cells from 3 biological replicates.

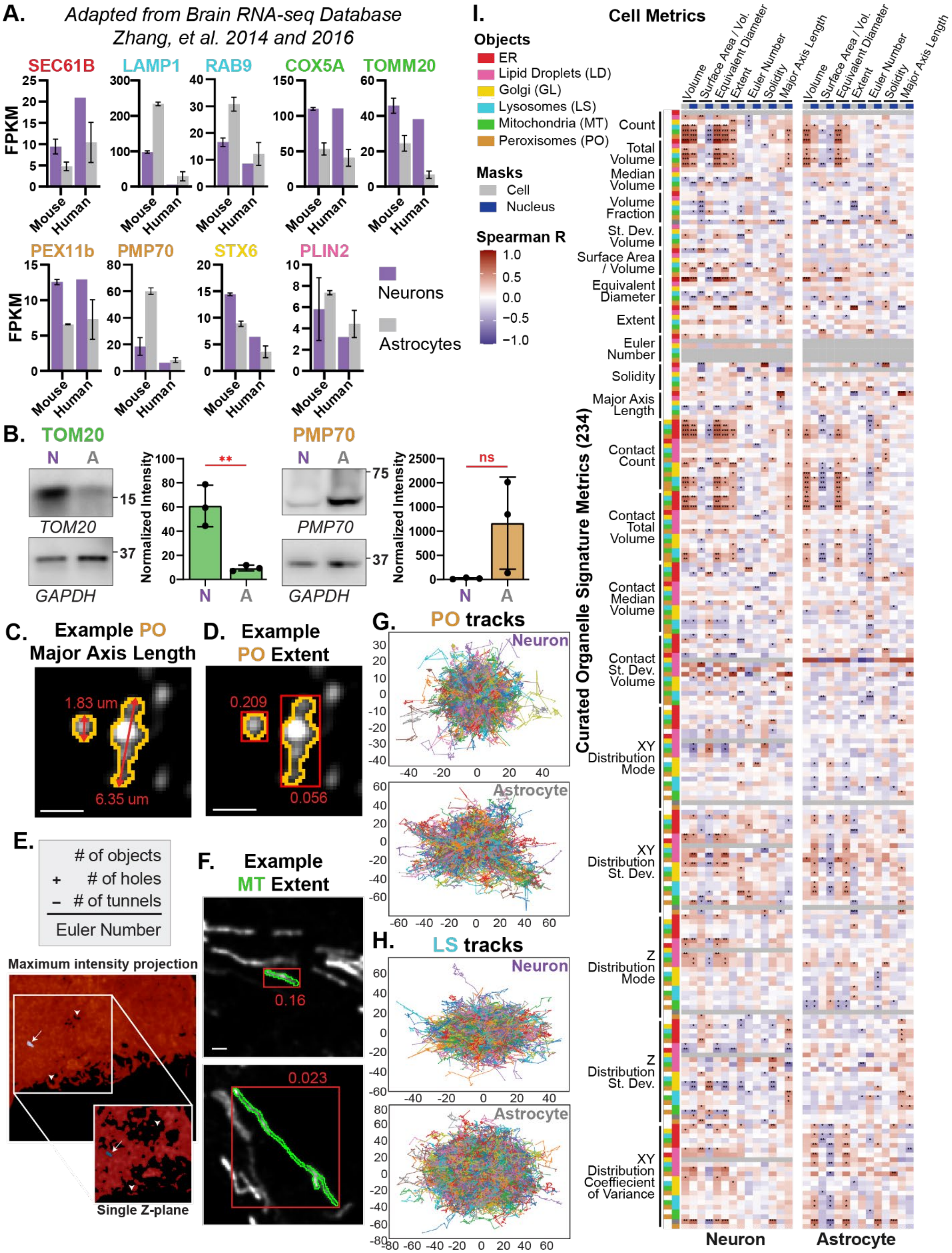

Figure S3. Neuron and astrocyte organelle morphology phenotypes corroborate findings from orthogonal approaches and correlate with some cell morphology metrics. A. RNA sequencing

results adapted from the Brain RNA-seq Database of stereotypical organelle markers; ER (SEC61B), lysosomes (LS; LAMP1 and RAB9), mitochondria (MT; COX8 and TOMM20), peroxisomes (PO; PEX11b and PMP70), Golgi (GL; STX6), and lipid droplets (LD; PLIN2). B. Quantitative Western blot analysis of canonical mitochondrial (TOM20) and peroxisomal (PMP70) proteins. C-D, F. Example intensity images illustrate shape measurements of individual organelle objects (outlines); scale bars are 1 μm. E. Euler number calculation and representative images displaying the components involved; images are maximum intensity projections or single z-plane of the ER segmentation; arrow indicates an example "hole" or space (highlighted in blue) within the interior structure of the ER; arrowheads indicate "tunnels" through the ER. G-H. Peroxisome (PO) and lysosome (LS) tracks from 2D time-lapse images plotted with the start of each track at the origin (0,0); axes represent distance in microns. I. Correlation heatmap of organelle signature metrics versus metrics of cell size and shape based on analysis included in Table S2; asterisks represent p-values.

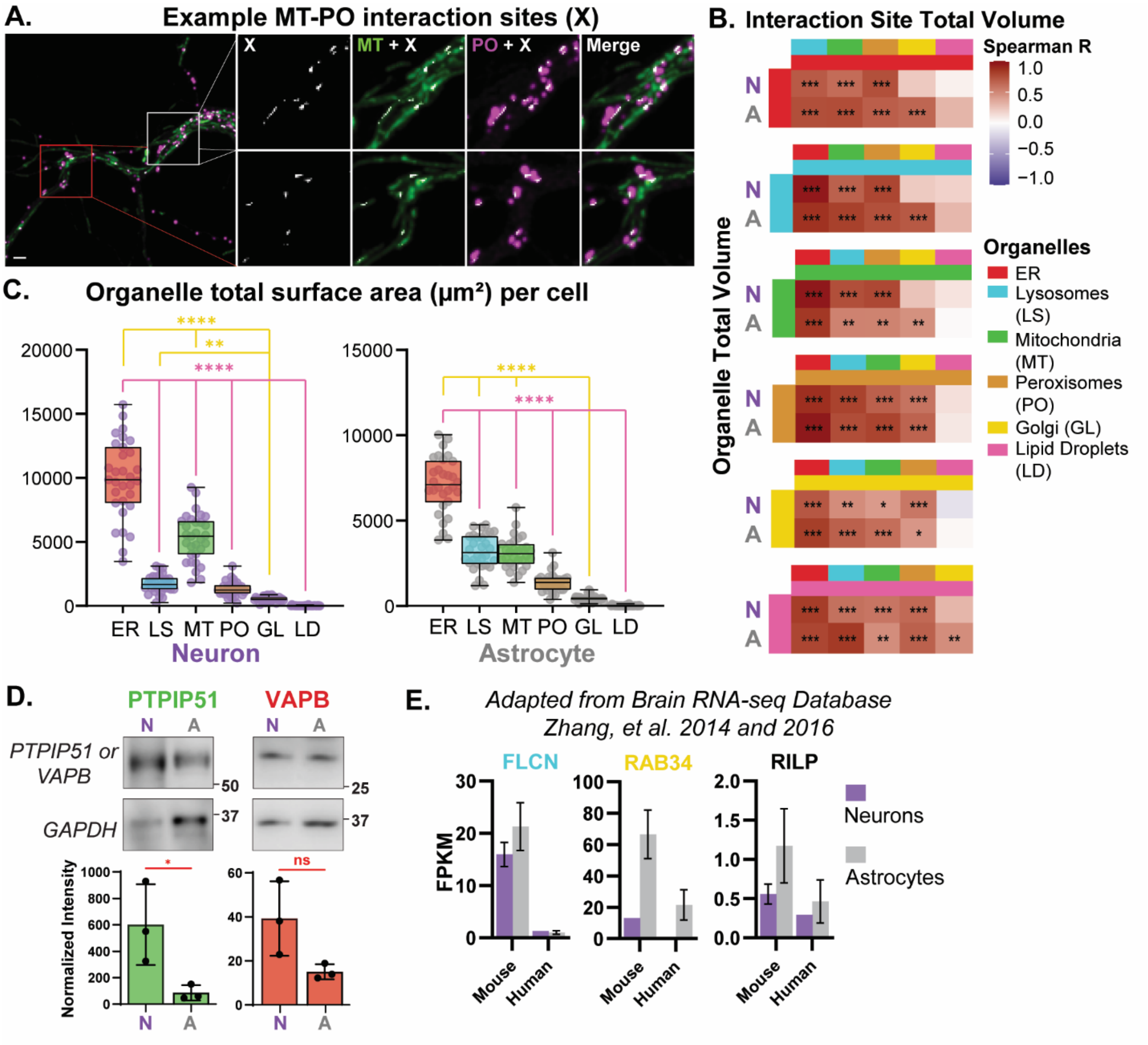

Figure S4. Organelle interactions and correlations to other organelle metrics, related to Figure 4. A. Example mitochondria-peroxisome (MT-PO) interaction sites (X; white) in a neuron overlayed onto the mitochondria (MT) and peroxisome (PO) intensity channels; scale bar is 1 µm. B. Heatmap of the Spearman R correlation coefficients for the pairwise interaction site total volume and the volume of their constituent organelles in neurons (N) and astrocytes (A); asterisks denote p-values from correlation analysis included in Table S2. C. Organelle surface area measurements from the full 3D dataset; asterisks indicate the p-value significance between LD (magenta) and Golgi (yellow) surface areas and the other organelles; data points represent single cells. D. Quantitative Western blot analysis of mitochondria (PTPIP51)-ER (VAPB) contact site tether protein levels from neurons (N) and astrocytes (A). E. RNA sequencing results adapted from the Brain RNA-seq Database of lysosome-Golgi (LS-GL) membrane contact tethers; FLCN (lysosome localized), RAB34 (Golgi localized), RILP (tether between RAB34 and FLCN).

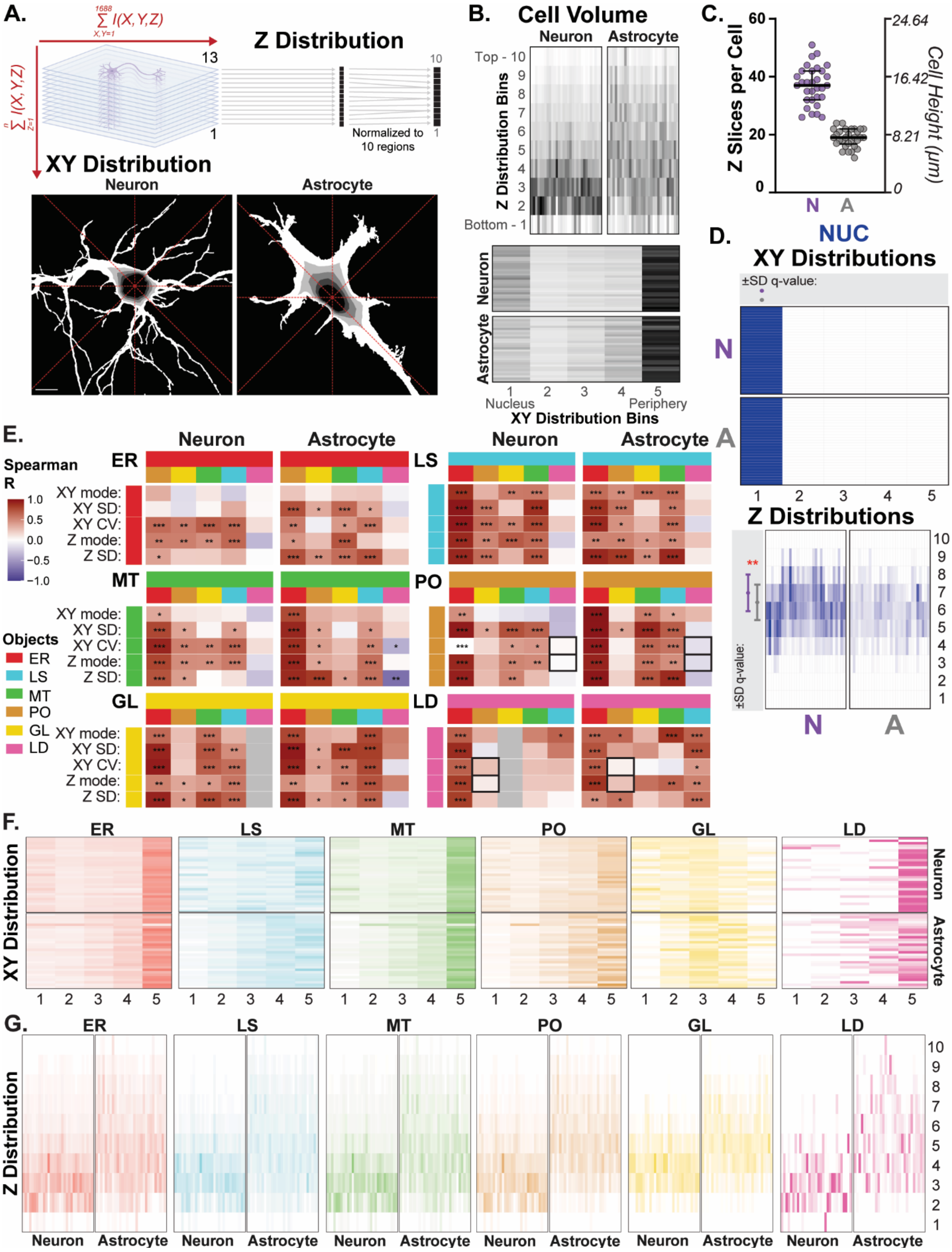

Figure S5. Subcellular distribution of organelles and organelle interaction sites, related to Figure 5. A. Schematic of XY and Z distribution measurements: To measure the XY distribution, segmented Z-

stack images were made into sum projections where the number of voxels containing an object (intensity; I = 1) was summed together for each XY position across all Z-planes (Z=1 → n). Using the resulting 2D cell and nucleus mask projections, the XY area was partitioned into five regions from the edge of the nucleus to the edge of the cell. The object volume per region was calculated across all Z-planes. Bottom: Greyscale images display example XY regions; red dashed lines denote the 8 equal radial sections used to calculate the coefficient of variance (CV) for each XY region separately; scale bar is 10 μm. To measure the Z distribution, segmented Z-stack images were made into sum projections where the number of voxels containing an object (I = 1) was summed together for each Z-plane across all XY positions (X,Y=1 → 1688). Right: Z-planes were separated into ten equal regions to summarize the organelle volume across the Z axis. B. Percent cell volume per XY and Z region; each column (Z) or row (XY) represents the values for individual cells; sample size and replicate information are summarized in Figure 1D. C. Median and interquartile range of neuron (N) and astrocyte (A) cell height; data points represent individual cell values. D. Heatmaps of the normalized nuclei volumes per XY and Z regions; dot plots above (XY) and to the left (Z) of the heatmaps summarize the mode (data point; asterisk perpendicular to the bars) and SD (error bars; asterisk parallel to the bars) distribution metrics per organelle. Each row (XY) and column (Z) in the heatmaps represents individual cells. Asterisks denote q-values from the comparison of neuron and astrocyte organelle signatures shown in Figure 2D; sample size and replicate information are summarized in Figure 1D. E. Heatmap of the Spearman R correlation coefficients for organelle interaction distribution metrics (x-axis) and their constituent organelle distribution metrics (y-axis); each row represents one type of distribution metric. Grey coloring indicates the correction was not calculated; asterisks represent correlation p-values from the analysis in Table S2. Standard deviation (SD), coefficient of variance (CV). F-G. Percent organelle volume per XY (F) and Z (G) region; data is the non-normalized equivalent to data shown in Figure 5A-B. Endoplasmic reticulum (ER), lysosomes (LS), mitochondria (MT), peroxisomes (PO), Golgi (GL), lipid droplets (LD), nucleus (NUC).

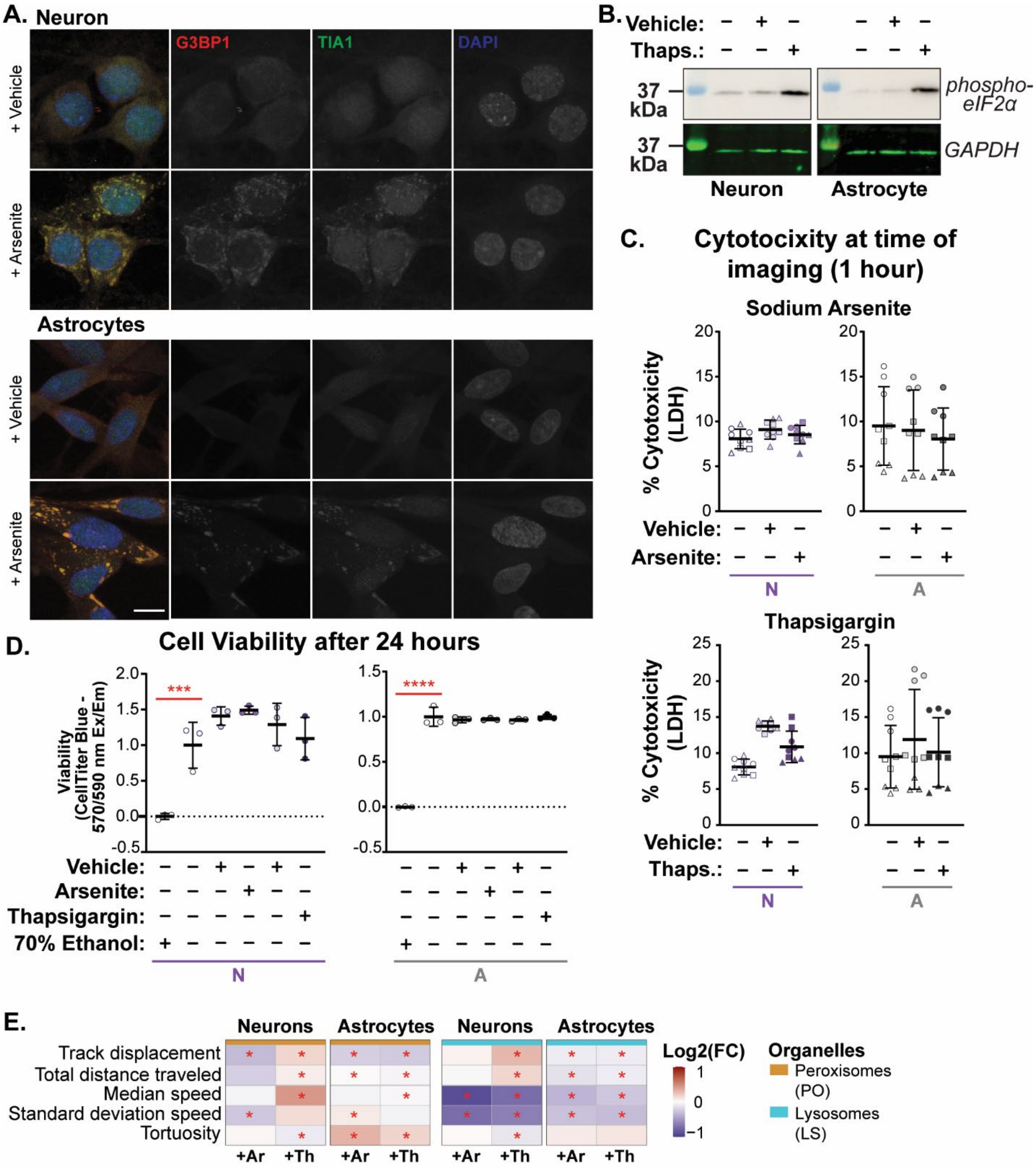

Figure S6. Sodium arsenite and thapsigargin exposure induce canonical oxidative and ER stress responses without a concomitant reduction in cell viability and cause alterations in organelle dynamics. A. Immunofluorescence microscopy of G3BP1/TIA1+ stress granules in neurons and astrocytes treated with 50 uM sodium arsenite for 1 hour; neurons: n=3 (43 cells), astrocytes: n=4 (37 cells); scale bar is 10 µm. B. Western blot of phospho-eIF2α in neurons and astrocytes treated with 25 nM thapsigargin for 1 hour; n=3. C. Mean ± SD percent cytotoxicity as measured by lactate dehydrogenase (LDH) levels following 1 hour of drug or vehicle exposure; asterisks denote significance as determined by one-way ANOVA; data points represent experimental replicates across biological replicates (shapes; n=3). D. Mean ± SD cell viability as measured by CellTiter Blue fluorescence 24 hours after exposing the

cells to sodium arsenite or thapsigargin for 1 hour; asterisks denote significance as determined by one-way ANOVA; data points represent biological replicates (n=3). E. Heatmaps of the $\log_2$ fold change (FC) of peroxisome (orange, left) and lysosome (cyan, right) dynamics from neuron and astrocyte control baseline conditions following a 1-hour sodium arsenite (+Ar) or thapsigargin (+Th) exposure; asterisks represent p-values from independent analysis.

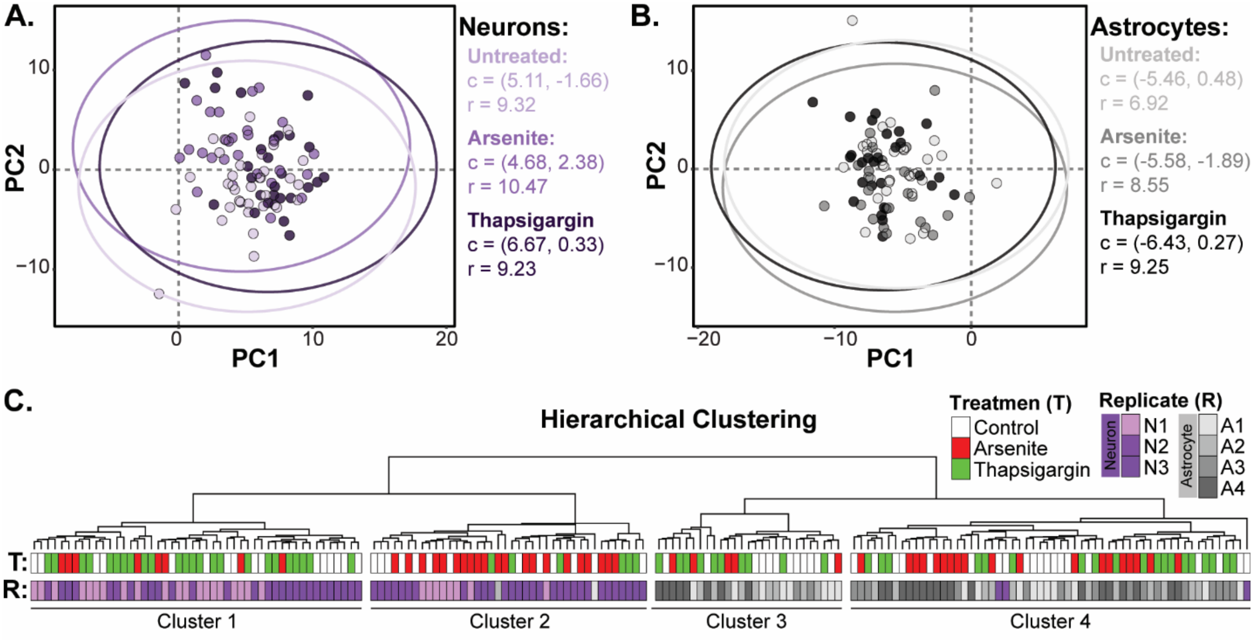

Figure S7. Stress-induced organelle changes are subtle compared to cell type differences, related to Figure 7. A-B. PC scores of neurons (A) and astrocytes (B) from Figure 7A separated by cell type; circles summarize the center of the data point for each condition (c) and the spread of the data points as displayed by ± 1.5 times the interquartile range (r). C. Hierarchical clustering of all neuron and astrocyte data across conditions revealed discernable clusters of neurons and astrocytes with three cells from neuron cultures and two cells from astrocyte cultures clustered with the opposite cell type; experimental replicates (R) are displayed as gradients of purple and grey; treatment conditions (T) are white, green, or red; sample size and replicate information are summarized in Figure 1D.

Supplemental Tables:

Objects	Category	Name	Description
Organelles; interactions	Morphology	Count	The number of unique objects per cell.
Organelles; interactions	Morphology	Total volume	The sum of all unique object volumes (skimage.measure.regionprops) per cell.
Organelles; nucleus	Morphology	Volume Fraction	The proportion of the cell volume occupied by the object; calculated by dividing the total object volume by the cell mask volume (skimage.measure.regionprops).
Organelles; interactions	Morphology	Median Object Volume (not ER)	The median volume (skimage.measure.regionprops) of all unique objects per cell.
Organelles; interactions	Morphology	Standard Deviation of Object Volume (not ER)	The standard deviation of volume (skimage.measure.regionprops) of all unique objects per cell.
Organelles	Morphology	Median Object Surface Area to Volume Ratio	The median surface area to volume (skimage.measure.regionprops) ratio of all unique objects per cell.<br><br>Surface area was estimated from a 2D surface mesh of the 3D object (skimage.measure.marching_cubes) using skimage.measure.mesh_surface_area. The ratio was computed in infer-subc.
Organelles	Morphology	Median Object Equivalent Diameter	The median equivalent diameter (skimage.measure.regionprops) of all unique objects per cell.<br><br>Skimage defines the equivalent diameter as: “The diameter of a circle with the same area as the region.” In 3D, this equates to the diameter of a sphere with the same volume as the region.
Organelles	Morphology	Median Object Euler Number	The median Euler number (skimage.measure.regionprops) of all unique objects per cell.<br><br>Skimage defines Euler number as: “For 3D objects, the Euler number is obtained as the number of objects plus the number of holes, minus the number of tunnels, or loops.”

Organelles	Morphology	Median Solidity	The median solidity (skimage.measure.regionprops) of all unique objects per cell.<br><br>Skimage defines solidity as: "Ratio of pixels in the region to pixels of the convex hull image."
Organelles	Morphology	Median Major Axis Length	The median major axis length (skimage.measure.regionprops) of all unique objects per cell.<br><br>Skimage defines major axis length as: "The length of the major axis of the ellipse that has the same normalized second central moments as the region."
Organelles; interactions; nucleus	Distribution	XY Mode	The concentric XY region with the most normalized organelle volume per cell. Normalized volume values were utilized as the frequency distribution to generate a histogram, and the mode was calculated from the resulting histogram.
Organelles; interactions; nucleus	Distribution	XY Standard Deviation	The standard deviation (SD) of the normalized volumes across the five concentric XY regions. Normalized volume values were utilized as the frequency distribution to generate a histogram, and SD was calculated from the resulting histogram.
Organelles; interactions; nucleus	Distribution	Z Mode	The Z region with the most normalized organelle volume per cell. Normalized volume values were utilized as the frequency distribution to generate a histogram, and the mode was calculated from the resulting histogram.
Organelles; interactions; nucleus	Distribution	Z Standard Deviation	The standard deviation (SD) of the normalized volumes across the ten Z regions. Normalized volume values were utilized as the frequency distribution to generate a histogram, and SD was calculated from the resulting histogram.
Organelles; interactions; nucleus	Distribution	XY Median Coefficient of Variance per Bin	The coefficient of variance (CV) of the volumes per XY region was calculated and summarized as the median CV value per cell.

Table S4: Curated Organelle Signature Metrics (234). Organelle signature metrics included in organelle signature analysis. Organelles: ER, lysosomes (LS), mitochondria (MT), peroxisomes (PO), Golgi (GL), and lipid droplets (LD). Interactions: the 15 pairwise interaction sites possible between each of the six organelles

Figure 2C	Figure S2C	Figure S2D	Figure S2E	Figure 7A/S7A-B
ER_count	ER_count	ER_count	ER_count	ER_count
LD_median_euler_number	LD_median_euler_number	LD_median_euler_number	lyso_median_euler_number	lyso_median_euler_number
lyso_median_euler_number	lyso_median_euler_number	golgi_median_euler_number	mito_median_euler_number	mito_median_euler_number
mito_median_euler_number	mito_median_euler_number	lyso_median_euler_number	perox_median_euler_number	perox_median_euler_number
perox_median_euler_number	perox_median_euler_number	mito_median_euler_number	LD_median_solidity	LD_median_solidity
LD_median_solidity	LD_median_solidity	perox_median_euler_number	golgi_median_solidity	golgi_median_solidity
golgi_median_solidity	golgi_median_solidity	LD_median_solidity	perox_median_solidity	perox_median_solidity
perox_median_solidity	perox_median_solidity	golgi_median_solidity	LDXgolgi_std_volume	nuc_XY_bins_hist_mode
nuc_XY_bins_hist_mode	LDXgolgi_std_volume	nuc_XY_bins_hist_mode	LDXgolgi_XY_bins_hist_mode	nuc_XY_bins_hist_stdev
nuc_XY_bins_hist_stdev	LDXgolgi_XY_bins_hist_mode	nuc_XY_bins_hist_stdev	nuc_XY_bins_hist_mode	nuc_XY_bin_CV_median
nuc_XY_bin_CV_median	nuc_XY_bins_hist_mode	nuc_XY_bin_CV_median	LDXgolgi_XY_bins_hist_stdev	
	LDXgolgi_XY_bins_hist_stdev		nuc_XY_bins_hist_stdev	
	nuc_XY_bins_hist_stdev		LDXgolgi_XY_bin_CV_median	
	LDXgolgi_XY_bin_CV_median		nuc_XY_bin_CV_median	
	nuc_XY_bin_CV_median		LDXgolgi_Z_slices_hist_mode	
	LDXgolgi_Z_slices_hist_mode		LDXgolgi_Z_slices_hist_stdev	
	LDXgolgi_Z_slices_hist_stdev			

Table S5: Variables removed from Principal Component Analysis. Variables from the curated list of 234 organelle signature metrics that were removed prior to principal component analysis, listed by figure. These variables had a standard deviation of 0 within the analyzed dataset.

Supplemental Data:

Data S1. FIJI macro script to measure neuron morphology.

```
// Define input and output directories
input = getDirectory("...");
output = getDirectory("...");

// Set batch mode
setBatchMode(true);

// Get list of files in the input directory
list = getFileList(input);

// Loop through each file
for (i = 0; i < list.length; i++) {
  // Check if the current file name ends with the suffix
  if (endsWith(list[i], "-cell.tiff") || endsWith(list[i], "-cell.tif")) {
    // Open the image
    open(input + list[i]);

      stackSize = nSlices;

      run("Z Project...", "start=1 stop=" + stackSize + " projection=[Max Intensity]");

      // setOption("ScaleConversions", true);
      run("Make Binary", "calculate black");
      run("8-bit");
      run("Skeletonize");
      run("Analyze Skeleton (2D/3D)", "prune=none");
      // run("Read and Write Excel");
      saveAs("Tiff", output + "MAX-skel_" + list[i]);

      imageTitle = getTitle();
      saveAs("Results", output + list[i] + "_skeleton_results.csv");
      run("Close");

    // Close the processed image
    close("*");
    run("Clear Results");
  }
}

// Exit batch mode
setBatchMode(false);
```
